# An Aggregated Data Integration Approach to the Web and Cloud Platforms through a Modular REST-Based OPC UA Middleware

**DOI:** 10.3390/s22051952

**Published:** 2022-03-02

**Authors:** Kaiser Habib, Mohamad Hanif Md Saad, Aini Hussain, Mahidur R. Sarker, Khaled A. Alaghbari

**Affiliations:** 1 Department of Electrical, Electronic and Systems Engineering, Faculty of Engineering and Built Environment, Universiti Kebangsaan Malaysia, Bangi 43600, Malaysia; p94749@siswa.ukm.edu.my or draini@ukm.edu.my (A.H.); 2 Institute of IR 4.0, Universiti Kebangsaan Malaysia, Bangi 43600, Malaysia; mahidursarker@ukm.edu.my (M.R.S.); alaghbari@ukm.edu.my (K.A.A.); 3 Department of Mechanical and Manufacturing Engineering, Faculty of Engineering and Built Environment, Universiti Kebangsaan Malaysia, Bangi 43600, Malaysia

**Keywords:** data integration, Internet of Things (IoT), Industrial Internet of Things (IIoT), Industry 4.0 (IR 4.0), intelligent system, OPC UA, REST, system integration

## Abstract

The Internet of Things (IoT) empowers the development of heterogeneous systems for various application domains using embedded devices and diverse data transmission protocols. Collaborative integration of these systems in the industrial domain leads to incompatibility and interoperability at different automation levels, requiring unified coordination to exchange information efficiently. The hardware specifications of these devices are resource-constrained, limiting their performance in resource allocation, data management, and remote process supervision. Hence, unlocking network capabilities with other domains such as cloud and web services is required. This study proposed a platform-independent middleware module incorporating the Open Platform Communication Unified Architecture (OPC UA) and Representational State Transfer (REST) paradigms. The object-oriented structure of this middleware allows information contextualization to address interoperability issues and offers aggregated data integration with other domains. RESTful web and cloud platforms were implemented to collect this middleware data, provide remote application support, and enable aggregated resource allocation in a database server. Several performance assessments were conducted on the developed system deployed in Raspberry Pi and Intel NUC PC, which showed acceptable platform resource utilization regarding CPU, bandwidth, and power consumption, with low service, update, and response time requirements. This integrated approach demonstrates an excellent cost-effective prospect for interoperable Machine-to-Machine (M2M) communication, enables remote process supervision, and offers aggregated bulk data management with wider domains.

## 1. Introduction

The Internet of Things (IoT) is an emerging global network affecting almost every domain by allowing the development of systems through intelligent devices and diverse application layer protocols according to user demand. Such sustainable growth of IoT technologies is also occurring in industrial application domains, namely the Industrial IoT (IIoT), which is leading industrial automation and communication infrastructures to undergo a profound advancement [1]. The advancement in industrial networking facilitated by the union of IoT and IIoT technologies reinforces the concept of the fourth industrial revolution (IR 4.0), standardized in (DIN SPEC 91345:2016-04 2016 IEC 2017) [2]. The IR 4.0 paradigm migrates current production systems into smart manufacturing, with a high degree of interconnection through intelligent networking and cognitive automation between the participating agents, and by shifting information into cloud-based applications for real-time data management [3]. This complex evolutional concept provides scalability to increase manufacturing flexibility, offer comprehensive information exchange, and enable virtual information representation in everyday internet-enabled devices as endpoints [4]. The evolution of the IIoT paradigm in industrial networks incorporates intelligent reconfigurable systems to process the production plant data flow based on various sets of requirements in a decentralized distributed manner. Distributed systems in shared cooperativity are holistic characteristics of modern factory systems, where data acquisition and information are processed locally in nodes of distant fields for a given industrial process. Hence, communication networks in such scattered applications enable the exchange of aggregated data among these nodes, and achieve mutual synchronization for controlling the overall production process.

However, industrial communication networks in the IR 4.0 context are currently a blend of both wired and wireless network technologies. Wired communications mainly include Modbus TCP/RTU, Fieldbus, EtherNet/IP, EtherCAT, TTEthernet, PROFINET, etc. [5]. Furthermore, the wireless networks are derived from the IEEE 802 protocol family such as IEEE 802.11 (Wi-Fi) and wireless personal area networks (WPANs) [6]. These infrastructures come from various manufacturers in different architecture and dissimilar hardware specifications, which often cause incompatibility issues and make the communication subsystems heterogeneous [7]. Collaborative integration of such heterogeneous systems to accomplish reliable data exchange and industrial processes management leads to communication interoperability issues among the protocols used and increased complexity of heterogeneity management challenges at various automation levels. Due to the different standards and language-based underlying models, data aggregation from heterogeneous sources at reduced latencies is a crucial challenge. Hence, the primary intention of this study is to incorporate an open-source unified model across multiple industrial networks to address communication interoperability issues at different automation levels and management of aggregated data from the heterogeneous sources in a standardized manner. Open-source technologies have been chosen over proprietary infrastructures, as they offer greater configuration options with necessary information, enabling the development of custom scientific tools and the recreation of physical artifacts without concerns about license expenditures. Incorporating open-source software is also economically beneficial and can provide, on average, 87% and even 94% of substantial economic savings involving both the open-source hardware and software development tools compared to the commercial proprietary equivalents [8].

To alleviate the issue in the communication network mentioned above, various IIoT protocols such as Modbus, PROFIBUS, and Distributed Network Protocol 3 (DNP3) can used named in the industrial domain to address network heterogeneity [9]. However, in the IR4.0-based context, the Reference Architecture Model for Industry 4.0 (RAMI4.0) [10] and Industrial Internet Reference Architecture (IIRA) [11] specify the reference models for next-generation factories to align with IR4.0 compliant standards. These reference models recommend Open Platform Communication Unified Architecture (OPC UA) as the major connectivity standard to implement underlying communication layers for interoperable information exchange between diverse industrial applications and to enable seamless information exchange across heterogeneous networks in the IR4.0 context [12]. According to the discussions above, OPC UA, as a promising IEC-62541 standard, has been chosen in this study to establish a unified platform among system components to aggregate heterogeneous data and automate interoperability among the system components in real-time. OPC UA is a vendor-neutral service-oriented-architecture (SOA) for industrial automation and Machine-to-Machine (M2M) communication [13]. It exhibits client–server or publisher–subscriber model-based communication, where data exchange takes place over encrypted TCP/IP through SSL, TLS, and AES [14]. OPC UA offers several combinations to map the client–server interactions, such as TCP-transported, binary-encoded, Hypertext Transfer Protocol (HTTP)-transported, Extensible Markup Language (XML)-encoded, and Simple Object Access Protocol (SOAP)-based services [15]. It defines object-oriented data descriptions through the group of nodes referred to as the information model. This reusable model allows the integration of other standards and provides transport and semantic interoperability to exchange information across the heterogeneous network [16]. Such an object-oriented information model provides great potentiality for OPC UA applications in various domains [17]. OPC UA exhibits the flexibility to adapt from embedded devices with small service sets to Windows, macOS, and Linux platforms with full functionalities [18].

IR4.0 compliant infrastructures experience syntactic interoperability to manage domain-specific data and semantic interoperability while exchanging data with shared meaning [19]. The OPC UA paradigm is lacking in the cross-domain era to interact with the external services. Hence unlocking the network capabilities of OPC UA is well demanded to share the resources with other domains. Besides, OPC UA applications are largely missing the granularity of web and AI technologies for developing adaptive platforms to virtually represent information and perform remote process supervision. OPC UA involves SOAP/TCP binary-based services whose complexity is incompatible with the resource-constrained devices that lead to energy and latency overhead [20]. Hence, OPC UA is not suitable for these devices without significant modification. Although OPC UA exhibits bulk data, it still lacks storage infrastructure. Small-scale traditional industries usually involve supervisory/monitoring systems for resource management. However, modern smart factories produce bulk data to process, which ask for structurally-accumulated storage systems for resource allocation [21]. The cloud paradigm is an excellent choice in this aspect, which offers data logging, location-independent bulk resource management, and ubiquitous content access. As addressing interoperability is a prerequisite requirement of IR4.0 systems, another intention of this study is to overcome the semantic and syntactic interoperability issues of the OPC UA-enabled applications in order to seamlessly integrate heterogeneous-aggregated data with other domains without any restrictions. Such phenomena can enable interoperable M2M communication from manufacturing execution systems (MESs) to enterprise resource planning (ERP) [22]. The path towards bridging OPC UA with other domains needs to involve a middleware protocol such as the Constrained Application Protocol (CoAP), Message Queue Telemetry Transport (MQTT), Advanced Message Queuing Protocol (AMQP), Representational State Transfer (REST), etc. [16]. Among them, the REST approach reaches almost every domain as a universal communication solution to harmonize communication and shows relatively better services in unstable networks [23]. Indeed, REST is a free and open-source software (FOSS) architectural style that can share information seamlessly with different agents and support open-source and proprietary systems. It is a protocol-independent, client–server architecture that exhibits a set of constraints for stateless application development. REST also provides uniform interfaces, mostly HTTP for resource representation via Uniform Resource Locator (URL) [24]. Such resource representations are compatible with web browsers and arguments are added with the request URL [25]. These features motivated us to use REST for establishing communications between OPC UA with different technologies.

Several proprietary solutions allow the REST interface to bridge OPC UA with the other domains. For instance, KEPServerEX [26] offers three predefined REST URLs to read, write, and browse OPC UA data. However, the browse service of KEPServerEX provides all the configured “tags” without node attributes. HyperUA [27] offers non-standard mapping by encoding OPC UA resources into the URL and provides an HTTP interface to access OPC UA node credentials for web clients. This approach asks for client-side HyperUA API implementation to interact with the server. The data FEED OPC suite [28] also incorporates the RESTful API to access OPC UA resources. These proprietary solutions lack customizability according to user requirements and configurability facilities to fabricate developed infrastructure with a deeper understanding and better limit awareness. Moreover, various adaptation approaches have also been taken with the OPC UA binary protocol to achieve the RESTful extension. Grüner et al. [29,30] proposed resource-oriented information models for RESTful sessionless extension of OPC UA. They enabled stateless service invocation and modified caching headers to reduce handshake signals and exchange stateless requests, and evaluated OPC UA over UDP to minimize the communication overhead. A similar concept was taken in [31], through the HATEOAS approach for interconnecting OPC UA nodes. However, these approaches do not meet the fundamental postulates defined by Fielding [24], and are therefore unable to integrate loosely-coupled distributed systems with other domains. Another study in [32], which follows the definition of REST postulates, proposed a Java OPC UA stack facilitating data integration between the OPC UA and HTTP/REST services. This approach provides access to the OPC information in a REST-based server to the RESTful clients. This approach also involves OPC UA sessionless service to achieve statelessness, does not have mapped interfaces to exchange OPC UA resources with the web domain. Although the OPC UA specification introduced a sessionless invoke method from Version 1.04 (OPC Foundation. Scottsdale, AZ, USA, 2018), most of the stacks still have not implemented the full functionalities for this version. Taking account of these studies, it can be commented that the protocol modification approach to making OPC UA adaptable with web technologies is not sufficient. OPC UA cannot be considered fully stateless due to it maintaining the established connection status with the clients, which is not compatible with the web domain.

A more appropriate approach will involve a gateway module in the middle to bridge the OPC UA with other domains. Incorporating REST is the best choice, as this paradigm is more compatible with the web domain than the other options, and can map the OPC UA resources into REST representation under the required specification. In [33], mapping of OPC UA server credentials as REST resources was proposed to investigate the conditions of heterogeneous industrial devices from monitoring applications. This approach involves a Java SDK-based OPC UA implementation that causes a processing burden for resource-constrained embedded devices. Furthermore, the solution involves proprietary protocols and enterprise applications to monitor the condition of heterogeneous field devices. In [34], an OPC UA-based gateway with an information server was proposed that integrates available production systems in an Industry 4.0 factory to allow supervision from the cloud, MES, or HMIs consumers. However, the functionalities of this gateway are limited to pre-defined templates that lack scalability and require custom configuration per cite. In another study in [35], an OPC UA server–client architecture-based gateway was proposed for the oil and gas industry. Here, the server collectively gathers Oracle simulator data and the client transfers these data to the NoSQL Oracle cloud server through the RESTful API to be store and share with enterprise applications. The configurability of this proposed system is limited by the Oracle IoT Cloud-based proprietary software, which lacks adaptability with other vendors. In [36], a declarative control concept through a REST-based OPC UA client decoupling approach was proposed to improve automation in a distributed system. Although this attempt enabled interoperability through the REST-based decoupling approach, the control functionalities were designed in a highly complex graph model through the OPC UA Browse-Service. Another attempt in [20] was made to integrate OPC UA with web technologies to address interoperability. In this approach, a lightweight middleware was proposed that maps the OPC UA resources into HTTP requests to monitor them from the RESTful web platforms, and also incorporates an HTTP frontend to execute the web platform requests to the OPC UA server. However, this attempt exhibits two crucial drawbacks. First, the absence of uniform interfaces lacks the scalability that is an important aspect of stateless communication. Second, mapping the OPC UA server responses into JSON or XML to achieve interoperability makes the situation more brittle.

Although the previous literature makes valuable contributions to RESTful extension of the OPC UA to integrate data with web technologies, it still exhibits certain limitations, such as the involvement of proprietary software limited by configurability, the mapping of server responses into JSON or XML-based representation causing processing burden for resource-constrained devices, the absence of uniform interfaces to integrate decentralized distributed system components, and the lack of experimental analysis to validate the proposed solutions. The proposed architecture overcomes these limitations through concrete strategies, and its implementation was validated by integrating with proprietary/open-source platforms and performing extensive performance analysis. To alleviate these limitations and achieve the research goals, the following questions are investigated in this research:

RQ1: What is the appropriate stack to develop an open-source vendor-neutral full-stack OPC UA platform for data aggregation in the heterogeneous distributed system?

RQ2: How do we extend the OPC UA functionalities with other domains through the uniform REST interfaces, in order to be compatible with the web and embedded devices?

RQ3: What is the most suitable open-source software framework to develop a web service client for remote supervision of the OPC UA resources and perform interoperable M2M communication with a low communication overhead?

RQ4: How do we integrate open-source cloud service functionalities with the OPC UA environment to achieve cost-effective aggregated bulk data management?

**Figure 1:**
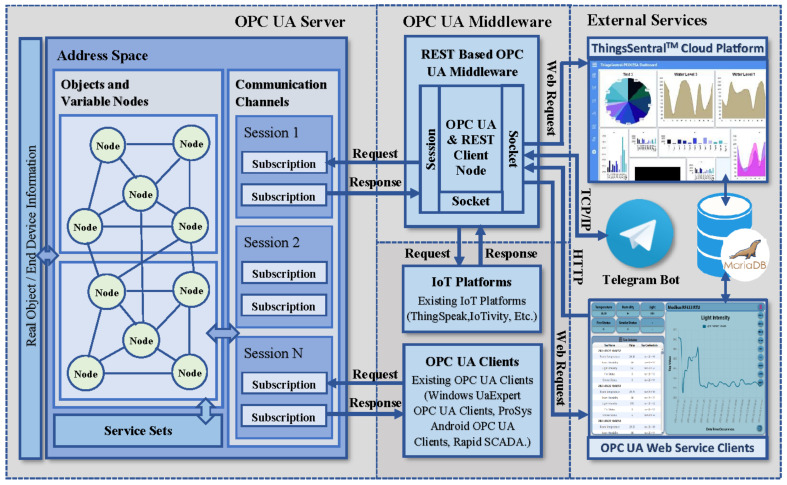
Architectural framework of the proposed system.

This study implemented an open-source cross-platform OPC UA server as the communication wrapper to abstract the underlying networks, aggregate data from the heterogeneous sources, and address technical and communication interoperability of distributed systems. The proposed framework also developed a cost-effective REST-based OPC UA middleware module to bridge the OPC UA resources with other domains through uniform resource representations to address syntactic and semantic interoperability. This inexpensive middleware module acts as an OPC UA client to obtain the server address space node credentials and map these collected resources into the prescribed web requests, in order to share the aggregated resources with the external services. As a proof of concept, we developed a RESTful standalone web service client and cloud platforms to collect the address space node credentials from the middleware, present these received resources virtually to perform remote process supervision, and enable aggregated bulk data management by allocating the received data in the cloud database. The middleware module can also share its resources with other existing open-source and proprietary platforms for data management, storage, and visual analysis. Furthermore, the Telegram Bot API functionalities were integrated with the middleware script to perform OPC UA tasks by invoking messages as commands from Telegram messenger. The overall architectural framework of the developed system is depicted in Figure 1.

Current industrial practice involves OPC UA-based propriety infrastructures, usually encapsulated for commercial purposes, and caters to the specific data transfer protocol. The proposed framework shows salient suitability in the IR4.0 context due to its simplified data flow, ubiquitous content accessibility, and open-source low-cost integration capability. The open-source architecture of the proposed system enables high design flexibility, allowing it to adapt from embedded devices with small service sets to Windows/Linux platforms and industrial automation systems with full functionalities. This scalable architecture has been tailored for engineering purposes, where the targeted users are students, researchers, and small and medium scale industries, to support seamless aggregated heterogeneous data management with several application domains for wider resources usability. The contributions of this proposed system summed up as:

1. We implemented an open-source vendor-neutral cross-platform OPC UA server to address communication interoperability and enable seamless aggregated data exchange across the heterogeneous distributed system at different automation levels;
2. We proposed an open-source REST-based OPC UA middleware to unlock OPC UA network capabilities to address semantic and syntactic interoperability to integrate aggregated data with other domains such as web, Telegram, and cloud services;
3. We developed a standalone web service client to receive OPC UA resources from the middleware, enable OPC UA address space remote supervision, and provide end-user application support to perform interoperable M2M communication;
4. We implemented a cloud platform named ThingsSentral^TM^ (http://thingssentral.io:443/) to collect and store the middleware data in a dedicated database for further visual data analysis, and aggregated bulk data management. The proposed framework also synchronizes Telegram messenger as the remote commander unit to perform remote supervision and notify users about the event occurrences;

The rest of this paper is structured as follows. The contextualization of the analysis framework and the systematic development procedure of the proposed system is described in Section 2. Section 3 validates the adaptability of the developed system in real-time scenarios by integrating the aggregated data from a proprietary DeviceXPlorer and Prosys OPC UA server to the web service and the ThingsSentral^TM^ and ThingSpeak cloud platforms. In Section 4, the performance analyses of the developed system deployed in a Raspberry Pi and Intel NUC PC are discussed in terms of platform resource utilization and operational time requirements. Finally, detailed discussions on the experimental results, the identified limitations, and future research scopes of the proposed framework are mentioned in Section 5.

## 2. Materials and Methods

In this section, the detailed development procedure of the OPC UA server to aggregate data from heterogeneous sources, a middleware module to integrate these data to the other domains, and a web service and cloud platform to collect, represent, and store these aggregated data are described with necessary figures, flow charts, and algorithms. Furthermore, the Telegram messenger as a remote commander and notification service is synchronized with the proposed framework. The materials involved and the methods used to develop the entire system are described below.

### 2.1. Proposed Framework

The participants of modern and IR4.0-compliant factories are dynamically interconnected through loosely coupled distributed systems, bounded by specific requirements to achieve a highly flexible manufacturing environment. Thus, addressing interoperability of multi-vendor systems is a prerequisite requirement of IR4.0-compliant infrastructure to aggregate data from the heterogeneous individual components. Besides, the ability to integrate different technologies is the paramount necessity of intelligent manufacturing systems to present information with the wider domain and enable interoperable M2M communication. Motivated by this realization, this study utilized the OPC UA paradigm to address the heterogeneity of underlying components, and utilized the REST paradigm to enable interoperability with web-based applications. The proposed system incorporates the IEC-62541 compliant OPC UA server as a communication wrapper whose information model stores and presents semantic information of the underlying networks through information contextualization. This feature enables the salient suitability of the developed implementation to aggregate data from distributed sources across the heterogeneous network. The proposed framework also introduces a middleware module to integrate aggregated resources of the OPC UA server with the other domains to address syntactic and semantic interoperability. This middleware module is an OPC UA client to collect heterogeneous resources from the server information model under the agreement of OPC UA specifications, also acts as a REST client to map the collected resources into web requests to share with the web and cloud services. As a proof of concept, a RESTful web service has been developed as the end-user application to collect the aggregated OPC UA server resources transferred from the middleware and present them virtually to perform OPC UA address space remote supervision over the internet. This web service client can also invoke requests to the OPC UA server through the middleware module to perform M2M communication without being compatible with the OPC UA standard. Furthermore, a cloud platform named ThingsSentral^TM^ was been developed and deployed at a publicly accessible address (http://thingssentral.io:443/) to store the aggregated OPC UA server resources transferred from the middleware in a dedicated database table structure for further data process analysis, both historically and graphically. Furthermore, the proposed framework also synchronized the Telegram messenger application with the middleware module to perform tasks as a remote commander and notify users about instantaneous event occurrences in an autonomous manner without continuous human investigation. The proposed framework was integrated with several open-source and proprietary platforms (as discussed in Section 3) to examine the adaptability and robustness of the developed implementation on real-time aggregated data management capability in both the education and industrial fields. The developed system can successfully integrate data from real-time heterogeneous infrastructures to several application domains with low platform resource utilization and operational time. The performance of the developed system was investigated through various experimental analyses deployed in a Windows-based Intel NUC PC and a Linux-based Raspberry Pi to examine cross-platform aggregated data integration capabilities with the other domains (as discussed in Section 4). These analyses were conducted by varying the number of OPC UA server address space nodes and connected clients in each platform, and the corresponding CPU, network, and power consumption rate were observed to achieve the best performance figures. The operational time requirements of the entire framework were also investigated, such as the average time required to update the server address space nodes, as resource update time; the average estimated time to collect the OPC UA server resources by the middleware, as service time; and the time to share these collected resources successfully with the external services, as response time. The remaining subsections discuss the detailed development procedure of the proposed system with the necessary information.

### 2.2. OPC UA Server Implementation

**Figure 2:**
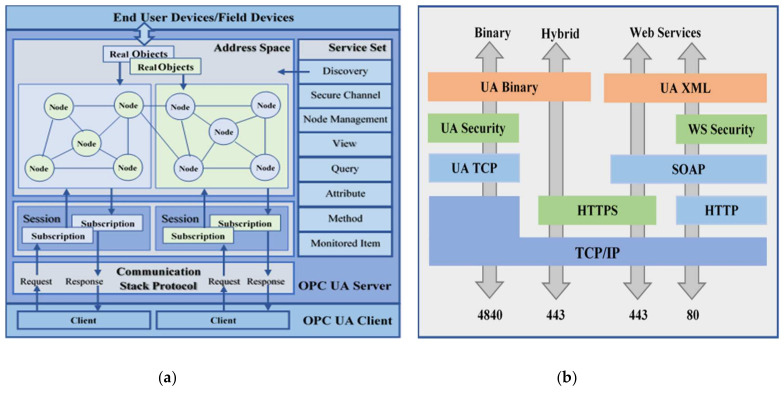
OPC UA server–client model: (**a**) structure; (**b**) data transport schemes.

The OPC UA server exhibits an information model in an object-oriented address space that uniformly represents objects via type definitions. This address space defines physical objects as nodes of different node classes and constitutes a graph of hierarchical nodes connected by the reference [37]. Nodes with necessary attributes are the key components to define information, where the address space allows access to these node credentials for OPC UA clients [38]. In this study, the end device measurements were updated in the OPC UA server dedicated address space variable nodes under the corresponding object node. The OPC UA clients can either obtain individual data or periodic updates from the address space through the established TCP/IP sessions [39]. Figure 2a illustrates the structure, and Figure 2b shows the data transport schemes offered by the OPC UA server–client model.

By default, the OPC UA Specification is assigned port 4840 for the UA Binary format, whereas the standard HTTP port 80 and secured HTTP port 443 are dedicated to the hybrid and XML-based data transport schemes. However, only one port can be assigned per communication channel for a selected data transport scheme, which is ideal for the firewall configuration [40]. The OPC Foundation has defined an extension of the Publish-Subscribe model (PubSub) for the OPC UA specification starting from Version 1.04 [41]. However, due to the lack of open-source cross-platform full-stack OPC UA Publish-Subscribe support, this study utilized the server–client model to develop the OPC UA environment. The OPC UA standard has been profusely applied in the industrial domain to interconnect multi-vendor equipment to address interoperability and harmonize the underlying communications. The OPC UA server uses a complex information model to represent information from these diverse system components in a structural manner. Such a complex structure requires an extensive understanding of this data model to access resources, creating difficulties for clients deployed in resource-constrained devices. Thus, the selection of an appropriate OPC UA protocol stack needs to focus on a customizable open-source implementation with economic maintainability and easier expandability in order to be compatible with dynamic systems, including embedded devices. Several proprietary and open-source OPC UA stacks under various licensing policies are currently available to implement a complete OPC UA server–client environment. The selection of the optimum protocol stack for this study was based on inexpensive full-stack platform-independent open-source lightweight implementation. Considering the key features of several OPC UA stacks mentioned in Table 1 [42], this study utilized the open-source Python-based FreeOpcUa stack [43] to answer RQ1. This platform-independent library module is flexible and scalable enough to be deployed even in embedded devices such as the Raspberry Pi. It supports multi-threaded operations to manage connections, sessions, and resources in separate threads. It is also able to exchange UA structure in the low-level interface and alter server resources in the high-level interface. This protocol stack offers custom address space generation and also provides necessary OPC UA agreement-based service sets to manage and share server resources with the OPC UA clients via both the node path and node id through established TCP/IP channels. Table 1 summarizes the key specifications of some OPC UA protocol stacks.

**Figure 3:**
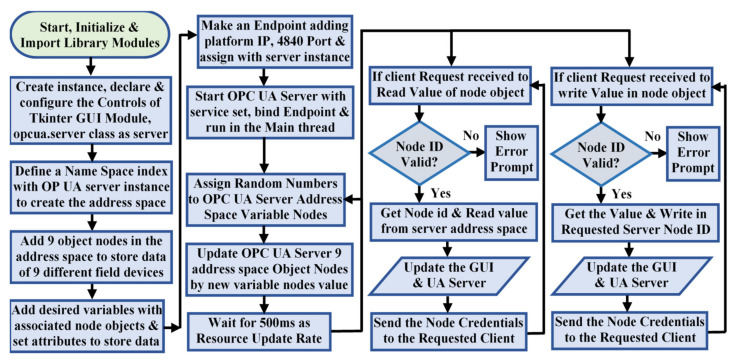
Process flow chart of the developed OPC UA server.

The address space located in the server is considered as an OPC UA core that stores data [44], while the service set is considered as another core to exchange this information with OPC UA clients [45]. The fundamental OPC UA entity is a request–response-based client-server model encoded in SOAP/XML/JSON/HTTPS/OPC UA binary format via standard TCP/IP [46]. This study involves a binary encoding format, as the embedded systems failed to support XML-based processing. The default prescribed format of the OPC UA server endpoint URI needs to be maintained as [opc.tcp://Platform IP Address: Port Number (default 4840)] to allow clients to discover the server in a network. The OPC UA server developed for this study collects data from the external sources periodically, updates the corresponding address space variable nodes with these data, handles the requests of the connected OPC UA clients, and also responds to the client requests through UA binary formatted response messages. All these phenomena need to be performed in a parallel manner at once without blocking any other code executions. Therefore, the Python multi-threading concurrency was utilized to support all these functionalities in different threads, while keeping the OPC UA core functionalities in the main thread. The process flow chart used to develop this OPC UA server is shown in Figure 3.

The developed OPC UA server code execution is categorized into three subsections. The first subsection establishes communication with the external sources and periodically collects information from these data sources. The second subsection updates these collected data into the corresponding address space variable nodes under the dedicated object. The third subsection manages communication and shares the address space node credentials with the connected OPC UA clients through the established TCP/IP sessions. This address space was configured with nine objects, with 63 variable nodes with a dedicated node id for each. End device measurements are periodically updated in these dedicated variable nodes. The algorithm designed to implement the code execution of these functionalities is mentioned in Algorithm 1.
**Algorithm 1.** OPC UA Server Development Algorithm.**Input:** Request messages from the OPC UA clients/middleware module.**Output:** OPC UA server endpoint URI and node credentials in UA binary format.1:  **function** StartOPCUAServer()2:    Construct server endpoint URI as *opc.tcp://platform IP address:port*3:    Create address space and define nine objects with associated variable nodes.4:    Start the OPC UA server with discoverable endpoint URI. 5:    **while** (*server.is_listening*) **do**6:       Call *Rand()* function to get periodic simulated random numbers. 7:       Update variable nodes by *node.set_value(variables)*8:       Wait *1000 ms* for the next iteration.9:    **end while**10:  **function** Listening(*Request*)11:    Listen continuously for the OPC UA client requests.12:    **while** (*Request.available*) **do**13:       Parse the NodeID, value, credentials from the request.14:       root *= server.get_root_node()*15:       Objects *= len(root.get_children())*16:       **if** (*Request == “Read”*) **then**17:          **for** (*i = 0;* Objects; *i++*) **do**18:             **if** (NodeID in *root.get_children*(i)) **then**19:                Name = *root.get_children()[i].get_browser_name()*20:                Value = *root.get_children()[i].get_value()*21:                Concatenate = Name + ‘=’ + Value22:                TagCredentials += Concatenate + ‘,’23:             **end if**24:          **end for**25:          Send TagCredentials to the client26:       **elif** (*Request == “Write”*) **then**27:          **for** (*j = 0;* Objects; *j++*) **do**28:             **if** (NodeID in *root.get_children*(j)) **then**29:                Write = *root.get_node(*NodeID*).set_value(*Value*)*30:             **end if**31:          **end for**32:       **end if**33:    **end while****Begin**34:  Start a thread calling StartOPCUAServer() to run the OPC UA server.35:  Start another thread calling Listening() to handle OPC UA client requests.**Finish**

**Figure 4:**
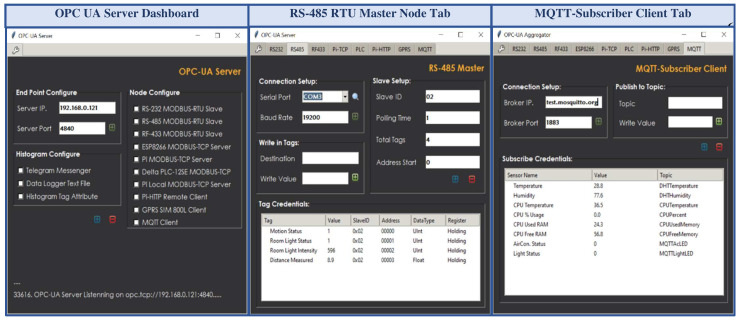
GUI application of the developed OPC UA server.

The OPC UA industrial standard interconnects equipment of different networks at different automation levels. It offers transport and semantic-level interoperability through the graph-based OPC UA data model. A Graphical User Interface (GUI) was designed for the OPC UA server application using the Python-based tkinter module [47]. This library is a standard, object-oriented, cross-platform Python built-in package that provides a low-level interface to create an interactive GUI for the Python script. Figure 4 shows the GUI application of the developed OPC UA server.

### 2.3. REST-Based OPC UA Middleware Implementation

The intention in implementing the REST-based OPC UA middleware was to establish a communication bridge between the OPC UA server with the external web service for remote process supervision, and with the cloud platform to store address space node credentials in a dedicated database server for further data processing, analysis, and decision making. The middleware was implemented with the same open-source Python FreeOpcUa stack used to develop the OPC UA server [43]. This study involved several methods to perform the OPC UA functionalities with the middleware through the server-offered service sets mentioned in Table 2.

**Figure 5:**
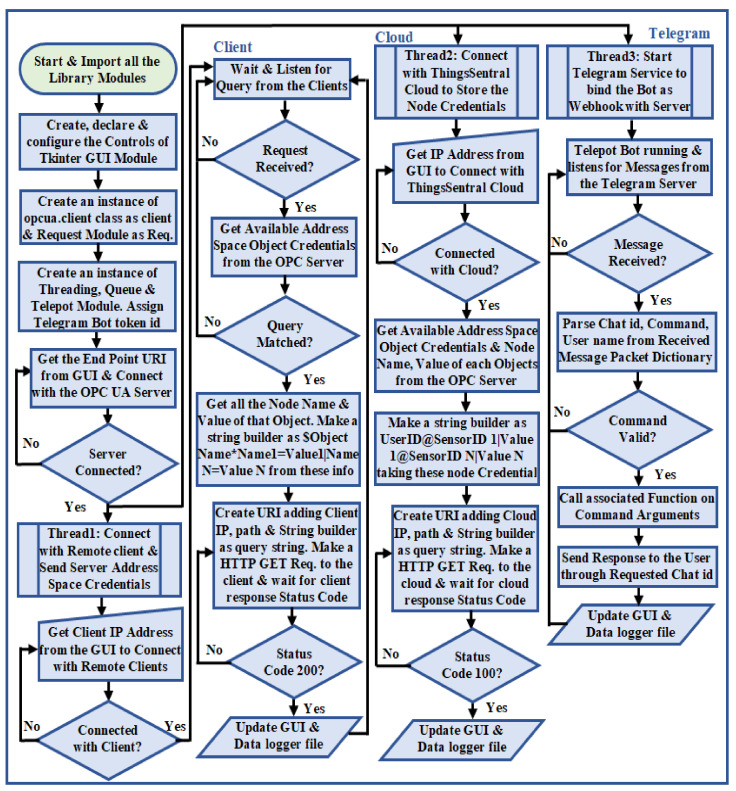
Process flow chart of the developed REST-based OPC UA middleware.

The entire middleware script was categorized into four subsections, where the code execution of each subsection was performed asynchronously in individual threads. The first subsection is responsible for opening the connection and establishing a TCP/IP session with the OPC UA server using Table 2, Method 1. It is also responsible for locating the external service availability in the network and obtaining user-requested object names from the middleware application GUI to share with the external services. The second subsection makes periodic requests to the connected server to obtain the address space node credentials by invoking Method 2 mentioned in Table 2, and checks the availability of user-queried object names in the received server object names. Mismatch among the user-supplied and server-received object names will produce an error message. A match between them instructs the middleware script to iterate through each object and collect the available variable node id of the corresponding object via Table 2, Method 3. For each node, the script collects the node name and corresponding value from the address space through invoking Method 5 and Method 6 as mentioned in Table 2. The third code execution subsection of the middleware script constructs individual web requests by concatenating these received node credentials as the request query string parameter in conjunction with the platform address, request path, and token id for the available external services according to the prescribed request format mentioned in Table 3. The flow chart to implement this REST-based OPC UA middleware functionalities is shown in Figure 5.

**Figure 6:**
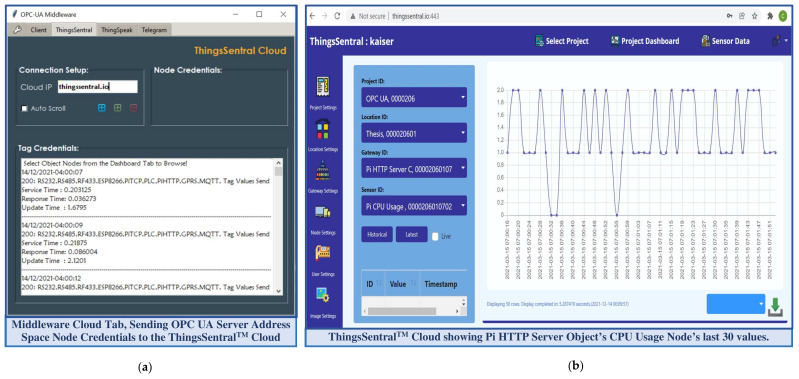
(**a**) Middleware application ThingsSentral^TM^ tab; (**b**) data monitoring in ThingsSentral^TM^.

A GUI was designed for this middleware application using the Python built-in tkinter library package to issue commands and configure the specifications of the external services graphically instead of by traditional console-based interaction [47]. The developed REST-based OPC UA middleware module can transfer the OPC UA server resources with several existing proprietary and open source IoT platforms, as most of these platforms, such as IoTivity sponsored by the Open Connectivity Foundation (OCF), SiteWhere from SiteWhere LLC, ThingsBoard from ThingsBoard™ umbrella, DeviceHive distributed under Apache 2.0 license, and ThingSpeak from MathWorks^®^, offers RESTful interface for data management, remote visualization, and rapid IoT-based application development [48]. This middleware also supports both proprietary and open-source OPC UA infrastructure, as well as industrial monitoring/supervisory software that offers reliable and seamless information exchange with different platforms without compatibility with IR4.0 or REST interfaces (as described in Section 3). As a proof of concept, the ThingSpeak platform [49] was configured to perform aggregated data integration phenomena through this developed middleware module. An average response time of 1.2 s was recorded for the middleware requests while sharing five address space variable node credentials to the ThingSpeak platform. However, the free subscription service of the ThingSpeak platform offers eight data fields to manage remote sensor data, one status, and three location fields to store device elevation, latitude, and longitude per channel. Furthermore, this cloud platform exhibits a 15 s interval rate to update successive data payloads in the JSON format. These are the crucial bottlenecking factors for a fast-paced real-time application to aggregate, store, and visualize bulk data streams. This study developed a cost-effective cloud platform named ThingsSentral^TM^ that can support aggregated bulk data management through the REST paradigm, which is not limited to eight data fields at a 15 s data transfer rate. As a proof of concept, all the 63 node credentials of the developed OPC UA server address space as 63 data fields were transferred periodically to the ThingsSentral^TM^ platform at a 0.5-s interval rate. The REST-based OPC UA middleware module facilitates sending these data through web requests, and an average of 0.45 s cloud response time was observed for the corresponding request, as discussed in Section 4.7. Figure 6a shows the ThingsSentral^TM^ cloud tab of the middleware application and Figure 6b shows the ThingsSentral^TM^ dashboard that represents ‘Pi HTTP Server’ address space object’s CPU usage variable node credentials.

This REST-based OPC UA middleware, which acts as an OPC UA client and makes periodic requests for the OPC UA server node credentials, was the approach taken to answer RQ2. It also acts as a REST client that sends web requests to the REST-aware-based web and cloud platforms to share the address space node credentials using the open-source Python requests module. This web request URL comprises an endpoint URI in conjunction with the HTTP request method. The endpoint URI is composed of the platform web address where the external service is running, the resource path as the root of the resource naming hierarchy, and the payload in the form of query string parameters. Both the web and cloud platforms incorporate a REST API that expects prescribed HTTP Get requests from the middleware, as mentioned in Table 3.

**Figure 7:**
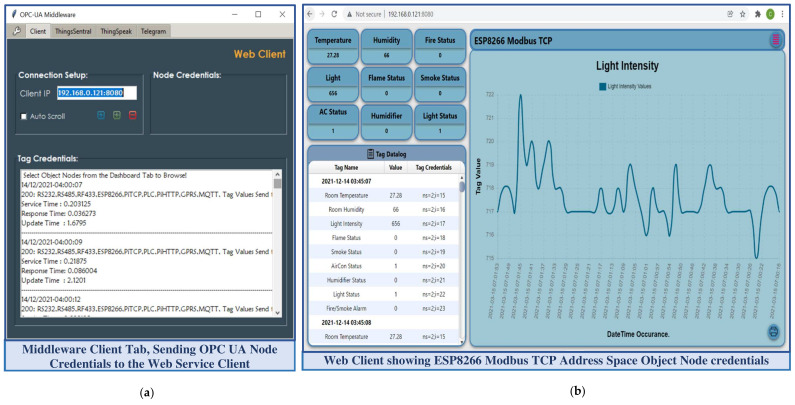
(**a**) Middleware application client tab; (**b**) middleware data monitoring in web client.

The middleware as an OPC UA client interacts with the server through the established communication stack to convey requests from web service clients, such as altering or synchronously accessing the server resources. It also periodically shares the server updates asynchronously as a REST-aware-based client with all the available instances of the ThingsSentral^TM^ cloud and web service clients present in the network. This study involved the HTTP GET method to share the OPC UA server node credentials with the external services through web requests. ‘OPC’ and ‘postlong’ are the request paths to make web requests to the web and cloud services, for which the REST APIs of these platforms are expected to receive data. The ‘&’ keyword in the request is used to join multiple parameters to share several data in the same request URL. After constructing the request URL, the middleware script sends the HTTP Get requests to these external services and waits for the corresponding response. Getting status code ‘200′ from these services as the acknowledgment of a successful request completion repeats the process. Figure 7a shows the web service client tab of the middleware application and Figure 7b shows the dashboard of the developed web service clients statistically representing the ‘ESP8266 Modbus TCP’ address space object credentials, as well a line graph taking the last 40 data of the ‘Light intensity’ variable node.

The last subsection of the middleware script deals with the Telegram Bot API service and binds the Telepot module in the individual thread. This module starts in a separate loop and continuously listens for the message in the telegram server. Upon a message uploaded in the Telegram server, the callback function of the Telepot module receives the packet dictionary from the server and extracts the chat id, user name, update id, and message as the command. This command request is examined with the scripted command arguments. A match between the user-supplied command request and the scripted command calls the associated function to perform the requested OPC UA server-specific task. The algorithm to implement the code execution of the middleware functionalities based on the process flow chart (Figure 5) is shown in Algorithm 2.
**Algorithm 2.** REST-based OPC UA Middleware Module Development Algorithm.**Input:** OPC UA Binary Data, User Query Request, and Telegram Commands.**Output:** Request URL to the web and cloud platforms. Notify for Telegram commands.01:  **function** ConnectServer()02:    **try** client = *Client.Connect(*ServerURL*)*
03:        **if** (client) **then break;**
04:    **except pass**05:  **function** MakeReq (*Platform, Address, Path* as arguments)06:    Get the root nodes as Query = *client.get_root_node().get_children()*07:    **for** (*i = 0*; len(Query.*get_children*(i)); *i++*) **do**08:        **if** (*client.get_object_node().get_children(i)* in Query) **then**
09:              Name =*Query.get_children()[i].get_browser_name()*10:              Value =*Query.get_children()[i].get_value()*; Concatenate1= Name+Value;11:              ClientBuilder += “$” + ServerObject(i) + “*” + Concatenate112:              CloudBuilder += Concatenate2; PCloudBuilder += Concatenate313:        **end if**14:        **if** (Platform *== ”web”*) **then**
15:              ReqURL = Address + “Path?” + ClientBuilder16:        **elif** (Platform *== ”ThingsSentral”*) **then**
17:              ReqURL = Address + “Path?” + CloudBuilder18:        **elif** (Platform *== ”PCloud”*) **then**
19:              ReqURL = Address + “Path?” + PCloudBuilder20:    **end for**21:    **try** Req = Get(ReqURL) **do**22:        **if** (*Req.Status == 200*) **then break**; 23:    **except pass**
24:  **function** StartTelegramWebHook()25:    **while** (Message available in Telegram server) **do**26:        Parse the message and store in chat_id, UserName, command variable.27:    **end while**28:    **if** (*command is valid*) **then**29:        Call associated function for the corresponding command request.30:        *Bot.sendMessage*(chat_id, Response)31:    **end if****Begin**32:  Start a thread calling ConnectServer() function to connect with OPC UA server.33:  Call MakeReq (*web, localhost, OPC*) to send OPC UA credentials to the web.34:  MakeReq(*ThingsSentral, thingssentral.io, postlong*) to send in ThingsSentral^TM^.35:  Call MakeReq(*Pcloud, Address, update*) to send credentials to the cloud. 36:  Start a thread calling StartTelegramWebHook() to run Telegram Bot.**Finish**

One of the significant features of the developed middleware is performing both read and write bidirectional communications without any prior knowledge of the underlying protocols. Furthermore, the ability to limit the number of communication signals simplifies the overall data flow across the system with a low communication overhead. According to the OPC UA agreement, exchanging resources need to establish a server–client connection through several service calls. Besides, multiple individual messages need to be exchanged between the server and client for having a single node attribute (value, type definition, encoding, etc.). This amount of communication signal increases while serving several clients through individual sessions that ask for additional network throughput and the platform CPU time to process requests and manage individual established sessions. This phenomenon creates a barrier for OPC UA applications running on resource-constrained devices. However, the middleware module, which acts as a single OPC UA client to collect the server resources through a single established TCP/IP channel and share them with the available external services in a stateless manner, can considerably reduce the number of communication signals and exchanged messages, the network throughput, and the platform CPU involvement. This scalable middleware module was tailored for engineering purposes, where targeted groups are students, researchers, and small- and medium-scale industries that require heterogeneous data management, analysis, and monitoring over the internet. The developed open-source REST-based OPC UA middleware module provides uniform interfaces that are fully compatible with web browsers, allowing an affordable aggregated heterogeneous data exchange with several domains and real-time bulk data management through cloud platforms. These features enable high design flexibility, allowing this module to be adapted from low-cost embedded systems with small service sets to the Windows, macOS, and Linux platforms and industrial systems with full functionalities to support cost-efficient seamless real-time operation in small and medium industries from the sensor to ERP level.

### 2.4. Web Service Client Development

**Figure 8:**
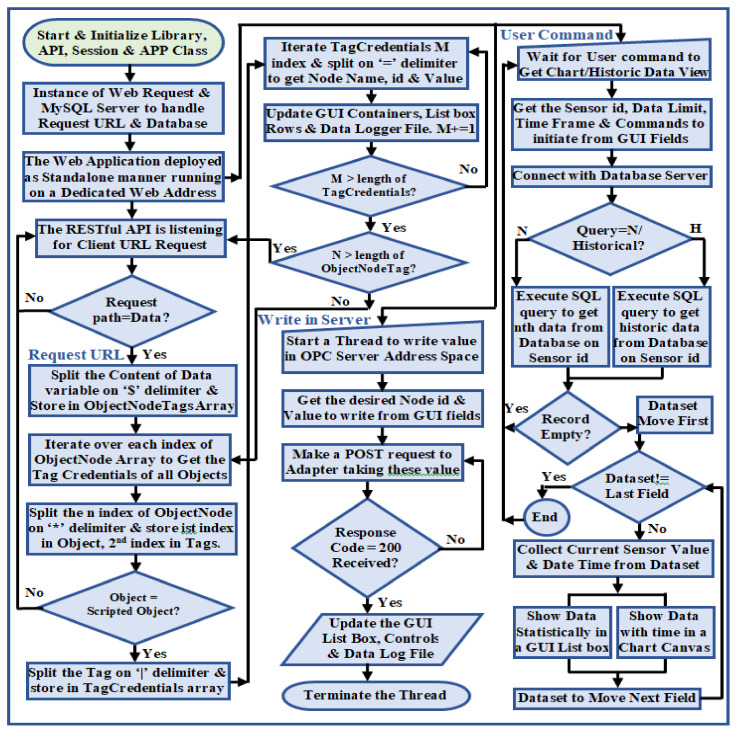
Process flow chart of the developed web service client.

The advantageous features of REST addressing interoperability as stated in Section 1, and the benefits of RESTful web services in industrial environments [25,29] motivated us to implemented a REST-aware-based web service client using the Xojo cross-platform application development tools to answer RQ3. Xojo facilitates the implementation of desktop, console, mobile, and web applications on multiple platforms such as macOS, Windows, Linux, Raspberry Pi, and iOS [50]. It utilizes HTTP 2.0 features to develop web-based applications that support almost every internet browser, and provides stable built-in controls to integrate database servers, HTML, and JavaScript-based third-party plugins with the application. Multi viewport meta tag scripting allows these applications to browse from any device with different screen ratios. The developed web service client is fully REST-aware-based; hence, it can be identified as a RESTful web service [51] that enables communication with system components in a stateless manner. Such stateless communication ensures sharing the same resources with several web service client instances asynchronously without maintaining the connection credentials indicating the requests from each instance are independent of any stored context. The process flow chart to develop this web service is shown in Figure 8.

**Figure 9:**
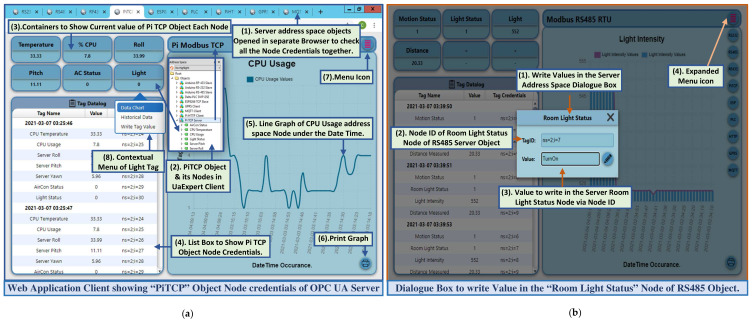
Web client GUI: (**a**) ‘PiTCP’ dashboard; (**b**) writing value in the server address space node.

The core code execution of this web service handles middleware requests, in which the code execution initiated from the user interface is performed in the session class. The user interface subsection takes user inputs, passes them to the Xojo core server to execute, and presents the server response in the corresponding browser table. This developed web service acts as a standalone REST server that continuously listens to the middleware web requests. A REST API was scripted, which handles middleware requests and extracts the entire query string parameter from the prescribed request for the ‘OPC’ request path only (as mentioned in Table 3). The API then parses this extracted query string on the ‘$’ delimiter to obtain the OPC UA server address space object, and splits again on the ‘*’ delimiter to extract the object name and corresponding node credentials. These node credentials are split later on the ‘|’ keyword, where the name and associated value are extracted by parsing each node credential on the ‘=’ delimiter. The web service script then updates the corresponding GUI controls with these parsed data. The web service offers a set of robust interface controls to monitor the node credentials visually without being compliant to the OPC UA standard, and allows synchronous requests to alter the server resources, for which the OPC UA server is bound to send responses facilitated by the middleware. Figure 9a shows a dashboard of the developed web service that represents the ‘PiTCP’ object credentials, and Figure 9b shows the dialogue box to write a value in the ‘Room Light Status’ node of the ‘RS485′ object.

The insight of the UaExpert client shows the developed OPC UA server address space with nine object nodes, where the ‘Pi TCP Server’ object exhibits six variable nodes (Figure 9a(2)). The developed web service contains nine web pages; each represents one OPC UA server address space object node credential toggled by a menu button (Figure 9a(7)). For instance, the PiTCP dashboard represents all the variable node credentials of the ‘PiTCP’ address space object in separate container controls, which are retrieved periodically from the middleware web request (Figure 9a(3)). Furthermore, the historical entity of these node credentials is shown in tabular form (Figure 9a(4)) and graphically through a line graph (Figure 9a(5)). Each GUI container utilizes a contextual menu (Figure 9a(8)) with three sub-menu options: Data Chart, Historical Data, and Write Tag. Before writing in an OPC UA server address space node, a valid node id (Figure 9b(2)) and value to write (Figure 9b(3)) need to be provided in the write dialogue box. The script then sends an HTTP POST request to the middleware taking this node id and value. Upon receiving a POST request, the middleware module parses the payload, communicates with the server, and writes the value passed for that node. The developed web service allows sharing the same application resources with multiple instances. This feature constitutes accessing a single application from several platforms without installing anything, while the resources remain common for all the instances. This web service exhibits server–client architecture-based request–response message exchange phenomena that allow it to deal with multiple clients asynchronously in a stateless manner.

### 2.5. ThingsSentral^TM^ Cloud Platform Development

**Figure 10:**
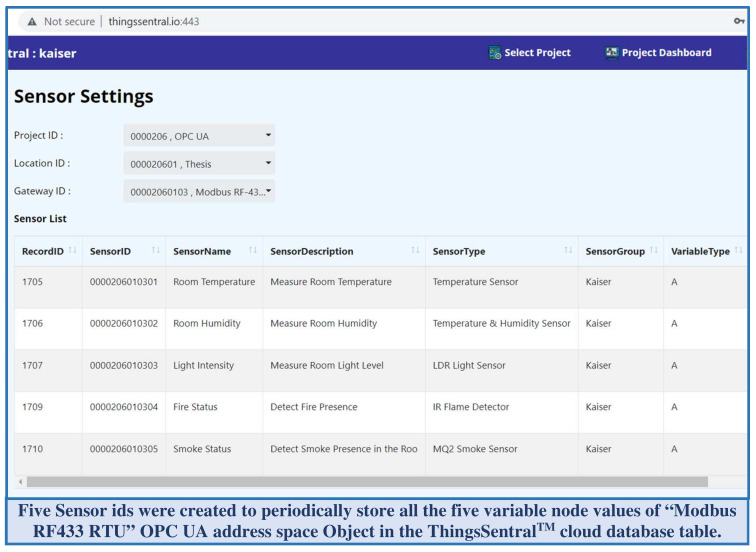
ThingsSentral^TM^ cloud platform Sensor Settings page.

This study developed a standalone REST-aware-based cloud platform named ThingsSentral^TM^ and deployed it on a publicly accessible web server with a dedicated web address (http://thingssentral.io:443) to answer RQ4. This platform was implemented using the Xojo application development tools, while cloud hosting was supported by an Apache webserver. ThingsSentral^TM^ provides facilities for inexpensive aggregated bulk data integration for remote clients structurally in a MariaDB database server, and also enables data analysis and graphical representation based on user queries for further investigation [52]. Storing data in the ThingsSentral^TM^ platform involves a preparation phase to create the desired number of sensor credentials. The ThingsSentral^TM^ GUI facilitates data management in the dedicated database table under an authorized user id and individual projects. Users need to create the project, location, and node credentials under a user id verified by the platform. The creation of the desired number of sensor credentials belonging to this node id constitutes a hierarchical tree-like structure to prohibit cross-referencing among the various user data. The ThingsSentral^TM^ platform assigns a unique sensor id for each credential as the identity to store and retrieve data from the associated database server. Figure 10 shows the sensor id created for this study to store the address space credentials in the ThingsSentral^TM^.

**Figure 11:**
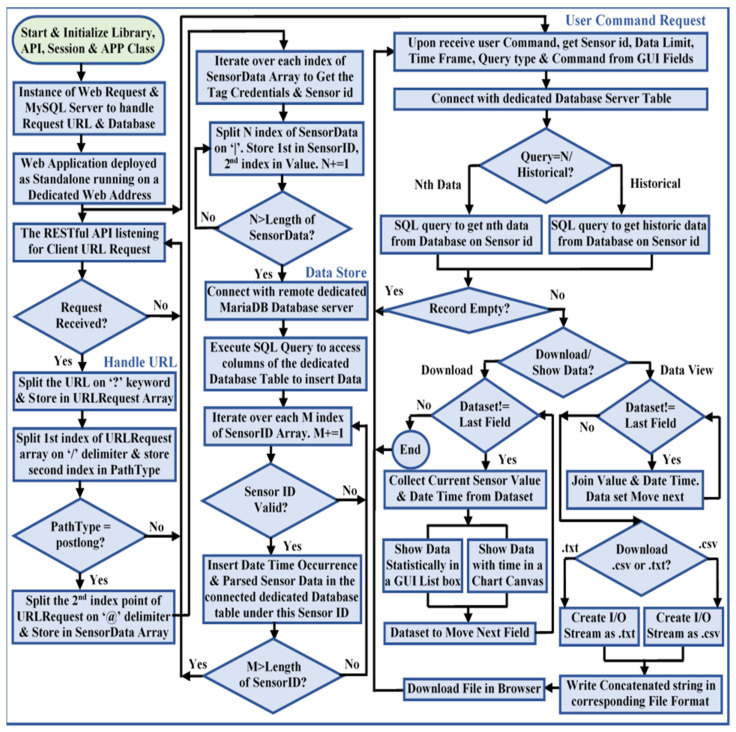
Process flow chart of the ThingsSentral^TM^ cloud platform.

ThingsSentral^TM^ allows the usage of the same application from multiple instances through two code subsections. The core code execution handles the HTTP Get-based prescribed web requests from the middleware, parses data from the request query string parameter for a valid request path, and stores the parsed payload in a database table under the assigned sensor id. The session-based subsection performs user input–output functionalities with the Xojo core server. The ThingsSentral^TM^ cloud platform does not rely on the established connection among the communicating devices; instead, it performs a request–response-based message exchange. The process flow chart of the ThingsSentral^TM^ cloud platform is shown in Figure 11.

**Figure 12:**
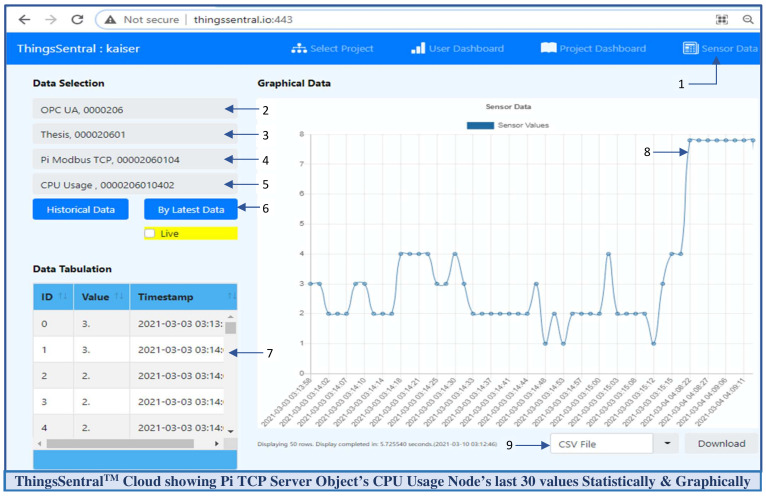
Data monitoring from the “Sensor Data” page of the ThingsSentral^TM^ cloud platform.

ThingsSentral^TM^ cloud platform exhibits short-lived, connectionless communication, and can deal with multiple clients asynchronously. A REST API was integrated with this platform and scripted such that it only allows HTTP Get-based prescribed URL requests, as mentioned in Table 3. For the request path ‘postlong’, this API extracts the request query string parameter on the ‘@’ delimiter to extract each node credential. These extracted node credentials are split on the ‘|’ keyword to obtain the value and sensor id. The script then makes a SQL query to insert these parsed data into the dedicated database under the corresponding sensor id. Several Arduino, Raspberry Pi, SIM800l, and NodeMCU-based gateway nodes were tested with the ThingsSentral^TM^ platform, and excellent data integration performance was observed. Figure 12 shows the last 30 stored ‘CPU Usage’ variable node data of the ‘Pi Modbus TCP’ address space object in the ThingsSentral^TM^. These data were retrieved from the ThingsSentral^TM^ database through the dedicated sensor id (0000206010402). The ThingsSentral^TM^ platform utilizes an individual node id (Figure 12(4)) to represent one OPC UA server object, under which the corresponding sensor ids were created. These node ids belong to a location id (Figure 12(3)) and project id (Figure 12(2)) whose information can be retrieved from the database for a given sensor id (Figure 12(6)). Also represent both statistically (Figure 12(7)) and graphically (Figure 12(8)) in the ThingsSentral^TM^ GUI.

ThingsSentral^TM^ is a scalable, and user-friendly cloud platform that offers rapid IoT application deployment for raw sensor data management for remote clients. Its GUI allows the creation of a customizable private dashboard for authorized supervision, or a public dashboard to monitor data globally. Data representation in the ThingsSentral^TM^ dashboard is performed graphically, facilitated by the several widgets, which are dynamically configurable according to user preferences under the date-time of event occurrence. This platform is currently under investigation by Universiti Kebangsaan Malaysia (UKM) engineering department students in several projects [53]. The algorithm designed to implement the web and cloud platform functionalities is shown in Algorithm 3.
**Algorithm 3.** REST-Aware-Based Web Service and ThingsSentral^TM^ Cloud Platform Development Algorithm.**Input:** Web request from the middleware. Sensor ID, Command Type (Statistics/Chart/View/Download), Query Type (Historical/N/csv/txt), From Date, To Date, Number to Query. NodeID & Value to Write.**Output:** Present OPC UA server node credentials and store in the MariaDB database. Command to write value in the OPC UA server node. Present all/historical database records graphically and statistically based on sensor id. 01:  **function** HandleURL()02:    Start the application in a standalone mannner and listen for the middleware URL request.03:    **while** (*Request.available*) **do**04:       Split request on ‘?’ and store in URLRequest array. Split the first index point of URLRequest on the ‘/’ and store in PathType variable. Store the last index point of the URLRequest in Data.05:       **if** (PathType *== “OPC”*) **then**
06:          Split data on the ‘$’delimiter and store in ObjectNodeTags array.07:          **for** (*i = 0 to length.*ObjectNodeTags, *i++*) **do**08:            Split ObjectNodeTags[i] on the ‘*’ delimeter & store Ist index in Object & 2nd index in Tags.09:            **if** (Object *in* ObjectList) **then**
10:               Split Tags on the ‘|’ delimiter & store in TagCredentials array.11:               **for** (*j = 0 to length.*TagCredentials*, j++*) **do**12:                  Split TagCredentials[j] on the ‘=’ delimiter.13:                  Store first index point of TagCredentials in Name and 2nd index in Values array.14:                  Update GUI, List Box, and Data Logger File taking these credentials.15:               **end for**
16:           **else break**17:         **end for**
18:      **elif** (PathType *== “postlong”*) **then**19:         Split Data on the ‘@’delimiter & store in SensorData array.20:         **for** (*i = 0 to length.*SensorData, *i++*) **do**21:            Split SensorData[i] on the ‘|’ delimeter & store 1st index in SensorID, 2nd in Value Array.22:            Make query as sql = “SELECT SensorID, SensorValue, DateTime FROM DatabaseTable”23:            rs = *mdb.SQLSelect(*sql*)*24:            **if** (rs not empty) **then**
25:               **while** (rs not reached to the last field) **do**26:                  *row.column*(*SensorID, SensorValue, DateTime*) = SensorID, Value, Time27:                  *mdb.InsertRecord(DatabaseTableName, row)*; rs.*MoveNext*28:               **end while**29:            **end if**30:         **end for**31:      **else pass**32:    **end while**33:  **function** WriteServerTag(*Value, id* as Arguments)34:     Collect middleware URL, id, value from the GUI and store in MiddlewareURL, NodeID, and Value.35:     Payload = {‘*Nodeid’*:id, ‘*value’*:Value}; response = *HTTP.POST*(MiddlewareURL, *data*=Payload)36:  **function** ExecuteCommand(*Command, QueryType, Sensorid* as Arguments)37:     Get Start Date, End Date, Limit from the GUI and store in From, To, Num. Connect with the MySQl Server.38:     **if** (QueryType == *Historical*) **then**39:         Collect historic data from the database for the given sensor id within the specified time and store in sql.40:     **elif** (*QueryType* == N) **then**41:         Collect N number of data from the database for the given sensor id and store in sql.42:      rs = *mdb.SQLSelect(*sql*)*43:    **if** (rs is not nil) **then**44:        **while** (rs not reached last field) **do**45:           Value = rs.*Field*(*SensorValue*); Time = rs.*Field*(*DateTimeAcquired*)46:           Join Time and Value on the ‘,’ and store in Concatenate Variable; Builder += Concatenate + EndOfLine47:           **if** (Command *== “Statistics”*) **then** *Listbox.AddRow*(Value, Time)48:           **elif** (Command *== “Chart”*) **then** *PlotGraph*(Time, value)49:           **else pass**50:        **end while**51:     **end if**52:  **function** UserRequest(*Command, QueryType, SensorID* as Arguments)53:     **if** (Command *== “Write”*) **then**
54:        Call WriteServerTag(*QureyType, SensorID*)55:     **elif** (Command *== “Statistics”*) **then**
56:        Call ExecuteCommand(*Statistics, QueryType, SensorID*)57:     **elif** (Command *== “Chart”*) **then**
58:        Call ExecuteCommand(*Chart, QueryType, SensorID*)**Begin**59:  Start a thread calling HandleURL() to collect data from the middleware request. 60:  Call UserRequest(*Write,Value,sensorid*) function to Write Data in the OPC UA Server address space.61:  Call UserRequest(*Statistics,Historical,sensorid*) to monitor sensor data historically in the application GUI.62:  Call UserRequest(*Statistics,N,sensorid*) to monitor latest N number of data in the application GUI.63:  Call UserRequest(*Chart,Historical,sensorid*) to monitor sensor data graphically in a historical manner.64:  Call UserRequest(*Chart,N,sensorid*) to monitor N number of selected sensor data graphically.**Finish**

### 2.6. Telegram Messenger as the Remote Commander Service

The proposed system synchronizes the open-source Telegram bot API with the middleware script to perform OPC UA server-specific tasks via messages as commands from the Telegram messenger. Configuring the Telegram bot with a unique bot token id was achieved by chatting with the ‘Bot Father’ in Telegram messenger. This token id is the bot identification in the Telegram server, which needs to be assigned when developing applications using this bot. The Python Telepot module was included in the middleware script to manage the Telegram bot API functionalities in a webhook PUSH API manner [54]. Webhooks are beneficial, as they proactively provide data when generated, instead of using a request–response-based mechanism [55]. The command arguments scripted to perform remote commander functionalities by the Telegram messenger are listed in Table 4.

**Figure 13:**
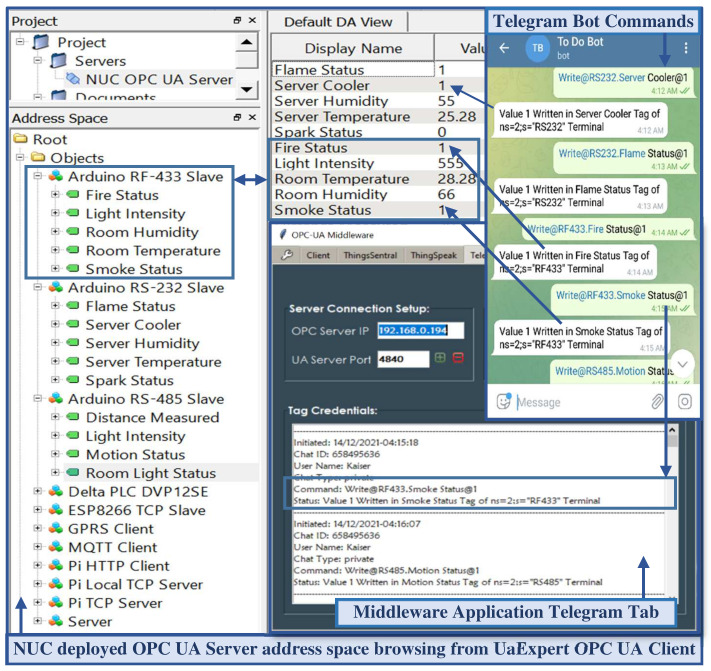
NUC deployed OPC UA server address space access from UaExpert and Telegram bot.

Messages issued from the Telegram bot are uploaded to the Telegram server as JSON data packet dictionaries with chat id, user name, message, update id, etc. Upon a message packet dictionary update in the Telegram server, the Telepot call back function collects and parses the packet and evaluates the parsed message as a command with the scripted command arguments. A match between them triggers the associated function to perform the corresponding OPC UA server-specific tasks and notify the command requestor through the chat id. Figure 13 shows the UaExpert OPC UA client and a command issued from the Telegram bot to alter the address space nodes.

Telegram service was incorporated for its cross-platform accessibility and mobile usability, which ensures faster mitigation action without continuous human investigation. Furthermore, incorporating the Telegram bot in the Telegram group allows access to the same resources with the broader community anytime, anywhere, over the internet.

## 3. Performance Evaluation of the Proposed System

**Figure 14:**
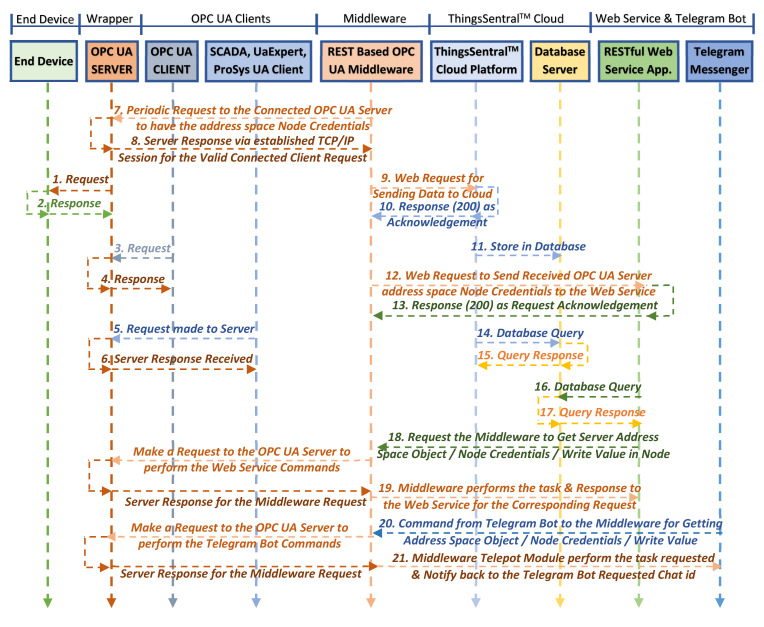
Data flow sequence in the entire system framework.

Figure 14 shows the data flow sequence in the entire system framework. Upon initialization, the OPC UA server creates an address space with the desired number of variable nodes, periodically calls the helper function to obtain sensor values (Figure 14(1)), and updates the values returned from the helper function in the dedicated address space variable nodes (Figure 14(2)). The connected OPC UA clients make requests (Figure 14(3,5)) to the OPC UA server to obtain these address space resources, and the server responds to the valid client requests (Figure 14(4,6)). The middleware, as an OPC UA client, also sends periodic requests to the OPC UA server (Figure 14(7)) to obtain these node credentials. Upon receiving the server response (Figure 14(8)), the middleware script then constructs web requests concatenating the received address space resources and sends the requests to the ThingsSentral^TM^ cloud (Figure 14(9)) and web service (Figure 14(12)). The ThingsSentral^TM^ cloud (Figure 14(10)) and web service (Figure 14(13)) receive and parse the payload from the request and send Response Code 200 to the middleware for successful request completion. ThingsSentral^TM^ cloud platform later stores these parsed data in a dedicated database table (Figure 14(11)). The GUI of both the web and cloud platforms allows the investigation of selected sensor data graphically and statistically by retrieving the data from the database server. It also offers to download the data in several formats (Figure 14(14–17)). The web service client (Figure 14(18)) and the Telegram messenger (Figure 14(20)) can request that the middleware perform OPC UA server-side tasks. Upon successful completion of the requested tasks, the middleware responds to these services for each request individually (Figure 14(19,21)).

**Figure 15:**
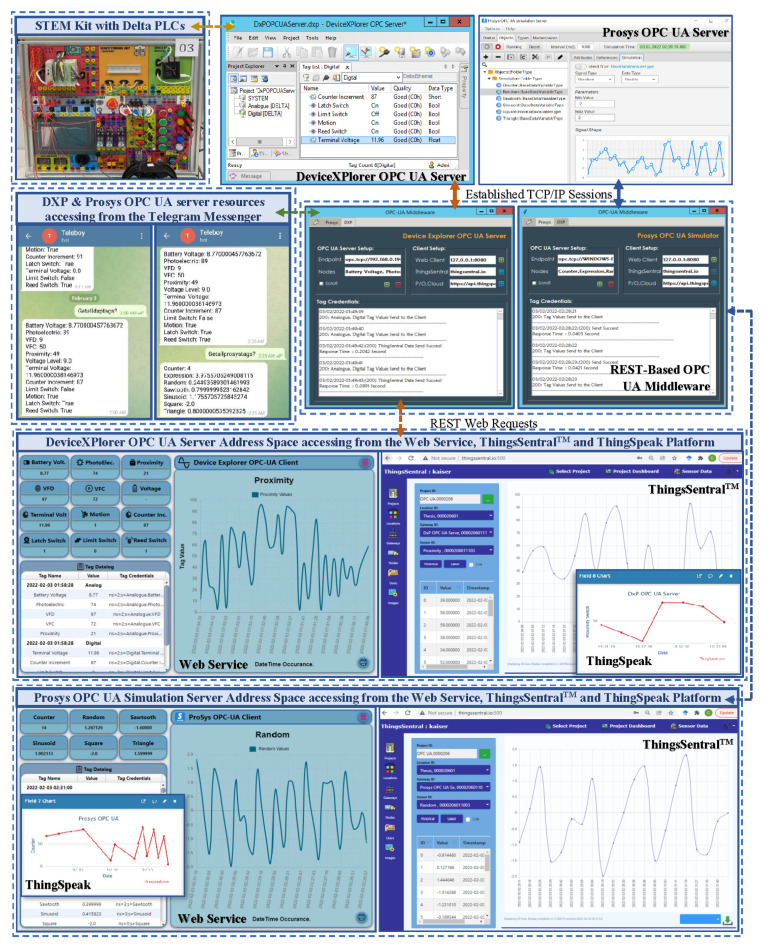
Evaluation setup of the developed system with proprietary and open-source software.

The OPC UA paradigm provides a discoverable endpoint URI to share the server address space credentials with the OPC UA clients. As an OPC UA client, the developed middleware module has sufficient realization of this paradigm to integrate with proprietary OPC UA servers such as DeviceXPlorer from Takebishi, NI LabVIEW from National Instruments, SIMATIC WinCC from Siemens, etc. Open-source OPC UA server simulators from Prosys, Integration Objects, Matrikon OPC, Unified Automation, etc. are also supported for OPC UA-based application testing, simulation, and validation purposes. Incorporating REST and the open-source design of the presented middleware module can feed the aggregated data from these OPC UA servers to the developed web service, the ThingsSentral^TM^ cloud, and several RESTful platforms such as ThingsBoard, IoTivity, ThingSpeak, etc. In this sense, different proprietary and open-source technologies can exchange information, which enables high design flexibility for affordable seamless aggregated heterogeneous data integration and real-time monitoring. As a proof of concept, the proprietary DeviceXPlorer OPC UA server (DXP) and the open-source Prosys OPC UA simulation server were integrated with this middleware module to aggregate data from heterogeneous sources. The ThingSpeak open-source IoT platform [49], the developed web service client, the ThingsSentral^TM^ cloud, and Telegram messenger were configured to integrate data from these aggregated OPC UA servers to the web domain. The open-source Prosys OPC UA simulation server address space constitutes an object class, with one node class of several simulation variable nodes such as counter, random, sawtooth, sinusoid, square, and triangle, with necessary attributes. A Science, Technology, Engineering, and Mathematics (STEM) trainer kit was integrated with the proprietary DXP OPC UA server deployed in a Windows-based Intel NUC PC. Two Delta DVP-12SE Programmable Logic Controllers (PLC) from this STEM kit were connected with the DXP OPC UA server to create a dynamic heterogeneous network. One of them included an analog extension module to interface with analog devices and communicate with the DXP server through the COM port via a Modbus RTU RS485 interfacing link (PLC-1). The other PLC interfaced with some discrete devices and communicated with the DXP server through Ethernet port via ModbusTCP protocol (PLC-2). Upon establishing the connections, two object nodes, named ‘Analog’ for PLC-1 and ‘Digital’ for PLC-2, were created in the DXP server address space. Each of these object nodes was configured with the necessary variable nodes for accessing and storing the PLC data through the corresponding memory register address. Running these servers provided discoverable an endpoint URI, [opc.tcp://{platform address}/OPCUA/SimulationServer] for the Prosys simulation and [opc.tcp://{platform address}/52240] the DXP server to share the semantic information of their corresponding address space node credentials with the OPC UA clients. Figure 15 depicted the evaluation setup for investigating the performance of the developed system with the proprietary and open-source software. The middleware periodically interacts with both the DXP and Prosys OPC UA servers as an OPC UA client to access their resources in two different threads through the established TCP/IP sessions in a synchronous manner. It also shares these collected server credentials asynchronously as a REST client with the web service, ThingsSentral^TM^, and the ThingSpeak platform through the prescribed request format, as mentioned in Table 3. A version of this middleware is currently being used as a gateway node in several IoT-based final-year undergraduate student projects at Universiti Kebangsaan Malaysia (UKM). Another modified version is currently supporting factory data management for several factories in Malaysia [56].

## 4. Performance Analysis and Results

The developed system was subjected to various performance test analyses such as platform CPU utilization, bandwidth consumption, and service, update, and response time requirements to investigate the implementation’s robustness and identify the best performance figures. These experimental observations are discussed below.

### 4.1. Testbed Setup

Each performance analysis was conducted by deploying the OPC UA server, OPC UA client, middleware module, and the web service clients on two platforms:

Platform 1: Windows Intel NUC PC (2.4 GHz Core i3 dual-core, 4GB DDR4 RAM);

Platform 2: Linux-based Raspberry Pi 3B+ (1.4 GHz Quadcore 64-bit, 1GB SRAM).

Furthermore, these performance analyses were investigated under two test cases:

Test Case A: Varying the number of OPC UA server address space variables from 1–63 nodes, and sharing these node credentials with a single OPC UA client;

Test Case B: Managing all the 63 OPC UA server address space variable nodes together and sharing these node credentials with up to 20 OPC UA clients.

**Figure 16:**
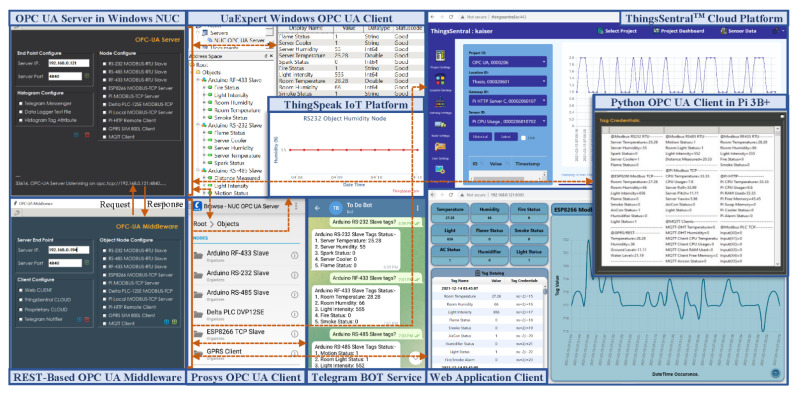
Testbed setup deploying the OPC UA server, middleware module, OPC UA clients, and web service in a Windows-based Intel NUC PC.

A Windows-based UaExpert [57] and an Android-based Prosys OPC UA client [58] were connected with the developed OPC UA server to investigate the cross-platform address space browsing. The experiments were carried out on a local network with 50 megabytes per second (MB/s) average measured bandwidth. A TP-LINK Archer C1200 router was configured to route the communication signals across the system. As the security policy of OPC UA affects its communication, the security function was disabled and no data encryption scheme was used. The measurement stability was ensured by repeating each experiment 10 times; the average of individual measurements has been reported. Figure 16 shows the testbed setup for conducting the experimental analyses.

### 4.2. Network Utilization Analysis

**Figure 17:**
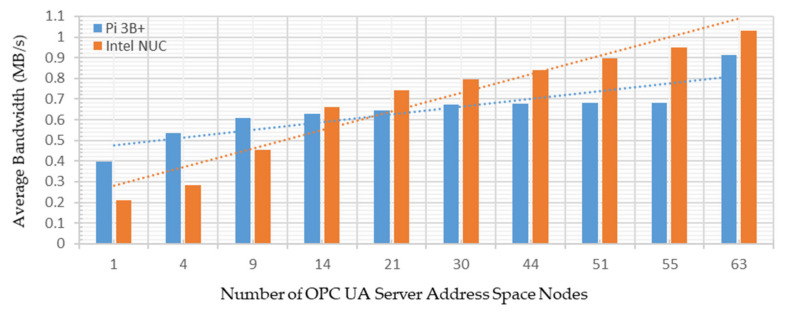
Average bandwidth utilization with varying the OPC UA server address space nodes.

This performance analysis investigated the average bandwidth consumed by the developed system deployed on both platforms under both test cases. The Python-based psutil net_io_counters method was used to measure network utilization in MB/s [59]. This method monitors system-wise network statistics and offers per-network information. The middleware was scripted such that it exchanged two pairs of messages in each call with the connected OPC UA server for a single address space node name and value. Thus, with the increased number of nodes to query by the middleware, the number of the exchanged message was increased, which required increased bandwidth. Figure 17 shows the average bandwidth utilized by the system under the first test case on both platforms.

**Figure 18:**
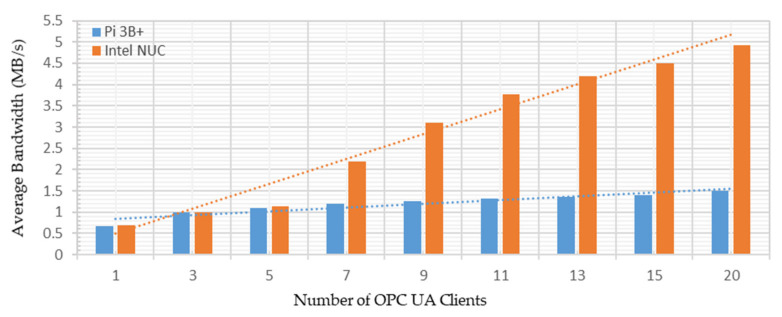
Average bandwidth utilization with varying the connected OPC UA clients.

From Figure 17, averages of 0.2121 MB/s and 0.3991 MB/s bandwidth were utilized to share a single address space node credential. These measurements were recorded as 1.0287 and 0.9116 MB/s to share all the 63 address space node credentials with a single OPC UA client on both platforms. Hence, averages of 0.8166 MB/s and 0.5124 MB/s additional bandwidth consumption were recorded to obtain all the node credentials, which were 1.6% and 1.1% of the available testbed network. Thus, with the increased nodes, bandwidth utilization also increases, showing a rising trend in Figure 17. Average network utilization under the second test case on both platforms is shown in Figure 18.

From Figure 18, averages of 0.6996 MB/s and 0.6662 MB/s bandwidth utilization were observed to share all the 63 address space node credentials with a single OPC UA client. These measurements were recorded as 4.9185 MB/s and 1.4968 MB/s while sharing 63 address space node credentials with the 20 OPC UA clients running on both platforms. Hence, averages of 4.22 and 0.83 MB/s additional bandwidth consumption were recorded to share all the node credentials with 20 OPC UA clients, indicating 7.8% and 1.7% of the available testbed bandwidth utilization. Thus, sharing node credentials with increased clients asks for additional bandwidth requirements, showing a rising trend in Figure 18. However, such small bandwidth consumption by the developed implementation is not a bottleneck factor. Higher network utilization may occur if multiple OPC UA servers send address space node credentials with several clients.

### 4.3. Power Consumption Analysis

**Figure 19:**
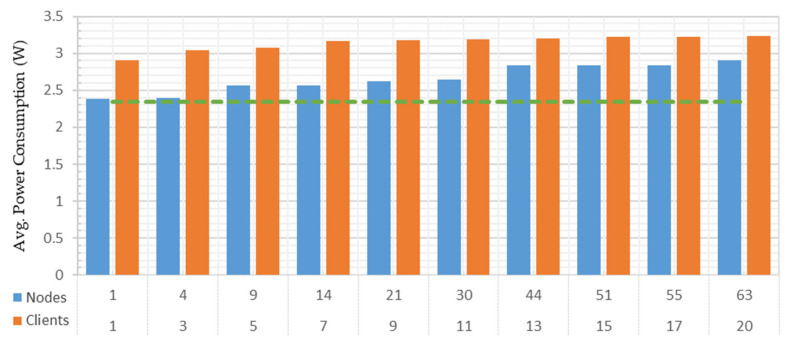
Average Pi power consumption with varying the OPC UA server nodes and clients.

In this performance analysis, the amount of power consumed by the Raspberry Pi 3B+-deployed OPC UA server and the middleware module was investigated under both test cases. The platform power consumption was estimated by connecting the INA219 current measuring sensor to the Pi’s main power supply. The Python INA219 library [60] facilitated extracting the bus voltage, current, and power consumption from this sensor through the Pi’s Inter-Integrated Circuit (I^2^C) interface. Before conducting the analysis, the Pi ran for a certain period without the server and middleware module. An average of 2.345691 watts of power consumption was measured, which was considered to be the Pi’s base power usage. Figure 19 shows the average power consumed by deploying the system on a Raspberry Pi 3B+ under both test cases.

The green dashed line in Figure 19 represents the Pi’s base power consumption. The blue bar represents the first test case, which showed an average of 2.3871 watts of power consumed to manage a single address space node. Sharing all the 63 node credentials with a single OPC UA client utilized an average of 2.9044 watts of power. Hence, 0.5588 watts of additional average power was consumed to manage and share all the address space nodes with a single client, compared to the Pi base power.

The orange bar represents the second test case, indicating an average power consumption of 2.9060 watts to manage and share all the 63 address space nodes with a single client. These measurements were recorded as 3.2312 watts to manage these available node credentials with 20 connected OPC UA clients. Hence, compared with the base power, an average of 0.3252 watts of additional power consumption was recorded in the second test scenario. However, although such small power consumption is not a bottleneck issue, it is crucial for powering the Pi with a secondary source that might not last for long in the case of continuous operation.

### 4.4. CPU Utilization Analysis

**Figure 20:**
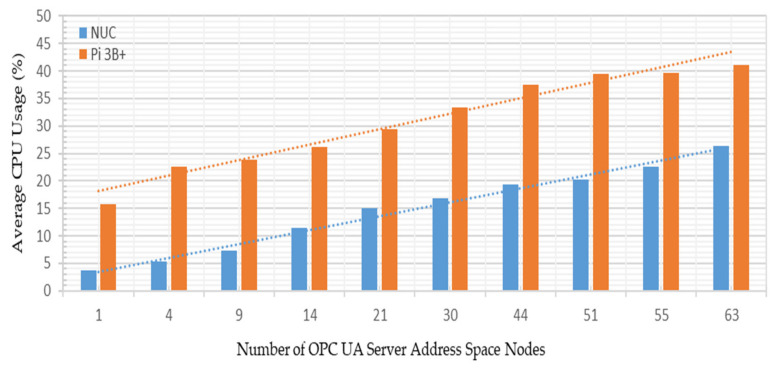
Average percentage of platform CPU utilized by the developed system with varying the OPC UA server address space variable nodes.

This performance test analyzed the CPU usage of the developed system on both platforms under both test cases. The Python-based psutil cpu_percent() method was used to investigate the percentage of platform CPU usage [59]. This method returns the CPU usage rate by measuring CPU times before and after a specified time interval. Figure 20 shows the average CPU usage by the system on both platforms under the first test case.

**Figure 21:**
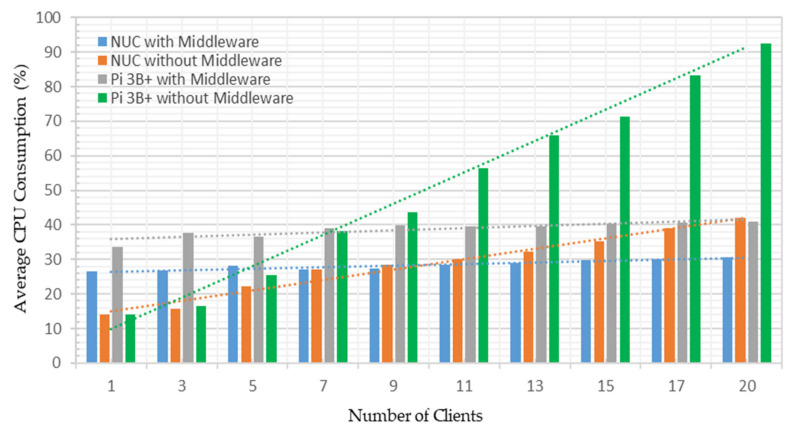
Average percentage of platform CPU utilized with varying clients.

From Figure 20, averages of 3.64% and 15.75% of the CPU were utilized, respectively, on both platforms to manage a single address space node and share it with a single OPC UA client. These average CPU utilization measurements were recorded as 26.39% and 41.02%, respectively, to manage and share all the 63 available node credentials with a single OPC UA client, indicating an additional 22.75% and 25.27% average platform CPU usage. An average of 14.62814% of additional CPU was utilized by the Raspberry Pi 3B+-deployed implementation compared to the Intel NUC PC. Hence, with increased nodes to manage, the average percentage of platform CPU usage also increases. Figure 21 shows the average CPU usage of the developed system deployed on both platforms with and without involving the middleware module under the second test case.

From Figure 21, averages of 14.02% and 14.10% of CPU were utilized, respectively, on both platforms with 63 node credentials by a single client without involving the middleware module. These measurements were recorded as 26.50% and 33.56% while involving the middleware. Hence, an additional 12.47% and 19.45% of the average platform CPU was utilized when deploying the middleware due to it performing several functionalities through individual threads. Averages of 42.01% and 92.49% platform CPU usage were recorded while serving 63 node credentials with 20 OPC UA clients, indicating an additional 28% and 78.38% of average CPU utilization on both platforms without involving the middleware. This high platform CPU usage rate can be decreased significantly involving the middleware module. From Figure 21, averages of 30.55% and 40.87% platform CPU usage were recorded to manage 63 address space nodes and share their credentials with 20 web clients through the middleware. This indicates 4% and 7% additional CPU utilization while serving 20 clients, instead of 42.01% and 92.49% CPU consumption on both platforms. This phenomenon shows a horizontal trend in Figure 21, with the CPU utilization rate remaining steady no matter how many web service clients must be served.

### 4.5. Resource Update Time Analysis

**Figure 22:**
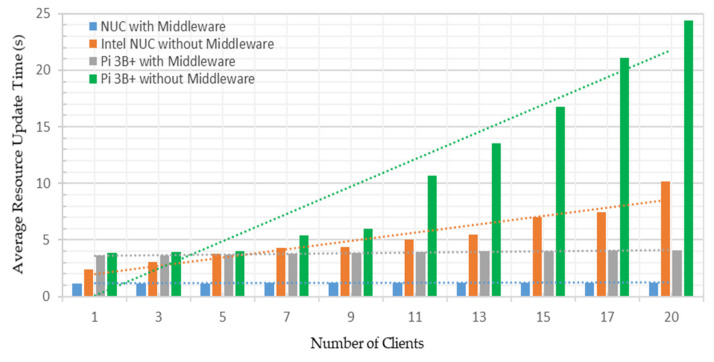
Average OPC UA server resource update interval time with varying clients.

In this experiment, the OPC UA server resource update rate was analyzed with and without involving the middleware module on both platforms. Experimental results showed that the average CPU and resource update interval rate increases slightly with the increased address space nodes. However, handling large-scale clients increases the server session management overhead, platform CPU utilization, and resource interval rate considerably. Figure 22 shows the average resource update time requirement for the increased clients on both platforms with and without involving the middleware module.

According to Figure 22, averages of 2.36 s and 3.86 s resource update time were observed on both platforms while managing all the 63 address space node credentials and sharing them with a single OPC UA client without involving the middleware module. These measurements were recorded as 1.14 s and 3.60 s, showing averages of 1.22 s and 0.26 s less resource update time required on both platforms to share the same amount of node credentials with a single web service client. However, managing all the 63 node credentials with 20 OPC UA clients requires an average of 10.16 s and 24.37 s resource update time, indicating 7.78 s and 20.51 s more resource update time required without involving the middleware module. The Pi-deployed server requires an additional 14.22 s more average server resource update time than the NUC one. These scenarios showed a gradual rising trend in the resource update rate for the NUC and an exponential rise for the Pi-deployed OPC UA server with the increased clients, as shown in Figure 22. The middleware module handles this situation by sharing node credentials with the available web service clients present in the network through a single established channel. From Figure 22, averages of 1.21 s and 4.07 s resource update time requirements were recorded on both platforms to manage all the 63 address space node credentials and share them with 20 web service clients, instead of 10.16 and 24.37 s while serving 20 OPC UA clients. This scenario showed a horizontal trend for both platforms, as shown in Figure 22, and improves the server resource update time requirement with the increased clients.

### 4.6. Middleware Service Time Requirement

**Figure 23:**
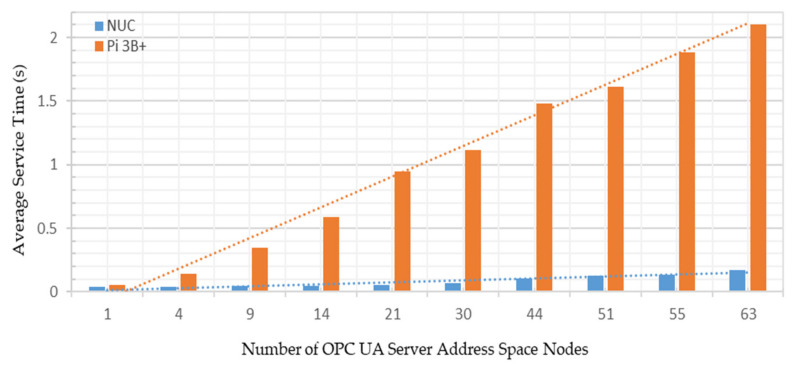
Average service time requirement with varying the server address space nodes.

This performance analysis investigates the average time required by the middleware to obtain the server address space node credentials as an OPC UA client, which is referred to as service time. Indeed, the service time is the interval between the instance of response received from a server to the time of the request made by an OPC UA client. Figure 23 shows the average service time required by the REST-based OPC UA middleware on both platforms under the first test case.

**Figure 24:**
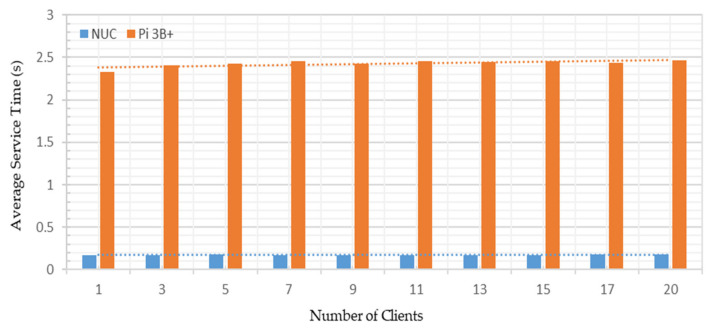
Average service time requirement with varying the number of web service clients.

Figure 23 shows averages of 0.0357 s and 0.0526 s of service time required by the middleware module to obtain a single address space node credential. These measurements were recorded as 0.1697 and 2.1051 s, respectively, on both platforms to obtain 63 address space node credentials. Hence, averages of 0.1339 and 2.0524 s additional service time were required to obtain all the node credentials by the middleware on both platforms. Thus, with increased nodes to query, the average service time also increases, showing rising trend in Figure 23. The average middleware service time requirement with varying web service clients on both platforms is shown in Figure 24.

According to Figure 24, averages of 0.1664 s and 2.3301 s service time required were recorded to obtain 63 address space node credentials by a single web service client. Sharing these 63 node credentials with 20 web service clients through the middleware required an average of 0.1779 s and 2.4660 s on both platforms. Hence, with the increase in web service clients, the average service time requirement remains almost the same on both platforms. This phenomenon shows a horizontal trend in Figure 24, no matter how many web clients are present in the network.

### 4.7. Middleware Response Time Analysis

**Figure 25:**
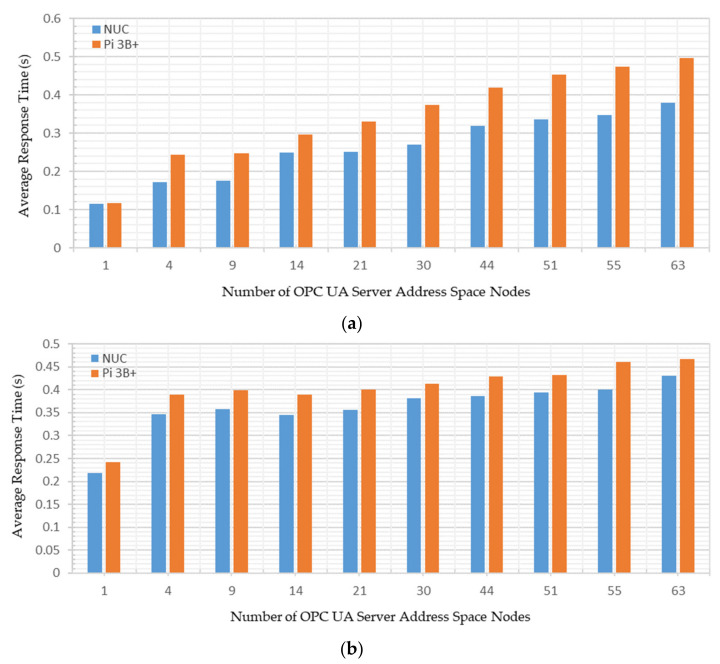
(**a**) Average web service response time with varying the server address space nodes; (**b**) average ThingsSentral^TM^ cloud response time with varying the server address space nodes.

This performance analysis investigated the average response time required by the REST-based OPC UA middleware requests to share the address space credentials to the web and ThingsSentral^TM^ cloud platform. Response time is the amount of time required to receive a response from the destination node as an acknowledgment of the source request. This measurement starts while sending a request and finishes upon receiving the response. This study used the Python request module to send requests and measure the absolute time difference between issuing the request and receiving the corresponding response [61]. The analysis results showed that sharing the increased number of address space node credentials through the web request requires additional response time, as it increases the length of the query string parameter. The graphical representation of the average web service client and ThingsSentral^TM^ cloud response times under the first test case are shown in Figure 25a,b.

From Figure 25a, averages of 0.1145 s and 0.1176 s of response time were recorded on both platforms for the middleware requests to the web service to share a single address space node credential through the query string parameter. These response time measurements were recorded as 0.2177 and 0.2428 s on both platforms while requesting the ThingsSentral^TM^ cloud service (Figure 25b). For sharing all the 63 address space node credentials through a single request, the average client response times were observed as 0.3794 and 0.4959 s (Figure 25a), and the average ThingsSentral^TM^ cloud response times were recorded as 0.4303 and 0.4665 s (Figure 25b), respectively, on both platforms. Hence an average of 0.2649 s client and 0.2125 s ThingsSentral^TM^ cloud additional response time was required to share all the NUC-deployed server node credentials. While sharing the Pi 3B+-deployed server’s available node credentials, an additional 0.3783 s client and 0.2237 s ThingsSentral^TM^ cloud average response times were recorded. However, such low response time is not a bottleneck issue for fast-paced real-time applications.

## 5. Discussion and Conclusions

This study demonstrates the concept of a cost-effective aggregated data integration approach to web and cloud platforms through a platform-independent open-source OPC UA wrapper and a REST-based OPC UA middleware module. The developed system showed tremendous prospects for dealing with cross-platform interoperability across heterogeneous networks and can perform real-time operation among different technologies with low platform resource utilization and operational time. The OPC UA binary-based solution overcomes communication interoperability through harmonization between different communication standards, can run in resource-constrained embedded devices, and can interconnect independent distributed system components according to the demanded specifications. Incorporating the REST paradigm in the middleware enabled stateless communication between the distribution systems and web platforms. Such a phenomenon enhances the OPC UA syntactic- and semantic-level interoperability to support seamless real-time information exchange among the open-source and proprietary infrastructures from several domains at once without any restrictions. This middleware also offers allows access and alteration of the OPC UA server resources by the web clients without any prior knowledge of OPC UA or compliance with this standard. Furthermore, the developed web service client is highly compatible with HTTP interfaces, which can be deployed on various platforms ranging from web browsers to embedded systems for performing interoperable M2M communications. The proposed framework shows salient suitability in the IR 4.0 context for its simplified data flow, ubiquitous content accessibility, and open-source low-cost integration capability. Performance evaluation in real-time scenarios validates that the developed framework can successfully integrate aggregated data from heterogeneous sources with the MES/ERP and open-source/proprietary platforms to enable a highly flexible manufacturing system in an open-source nature. The open-source design paradigm of the developed implementation offers high configurability to customize the system at any time without concerns about license expenditures. This also enables high design flexibility, from embedded devices with small service sets to Windows/Linux platforms and industrial automation systems with full functionalities. Besides, such open-source architecture provides the facilities to fabricate the developed infrastructure with substantial cost savings, with a deeper understanding and better limit awareness than commercial proprietary equivalents. According to the experimental results, network and power utilization by the developed implementation were observed as bottlenecking issues on either platform under both test cases, and can be considered negligible. However, performance analysis indicates that the Raspberry Pi-deployed implementation requires comparatively higher platform resource utilization and service and response times than the Intel NUC due to its hardware limitations. With increased nodes and clients to manage, the average Raspberry Pi CPU utilization also increases gradually. This situation was significantly improved by the middleware, which acts as a single OPC UA client to collect server address space resources through a single established TCP/IP channel with low service time, and shares it with the external services as a web request with low response time. The middleware module considerably reduces communication signals, the number of exchanged messages, network throughput, and platform CPU involvement, while performing overall system functionalities. The web service client running in a local network exhibited a lower response time than the ThingsSentral^TM^ cloud platform deployed in a remote web server. This additional operational time required to collect all the OPC UA server node credentials and share them with the ThingsSentral^TM^ is not a bottlenecking issue to performing real-time fast-paced operations. However, the experimental analysis found that the Raspberry Pi-deployed implementation with full functionalities and serving large-scale OPC UA clients in the same platform utilizes a higher CPU percentage than the Intel NUC PC-deployed infrastructure. The CPU times to update all the OPC UA server address space nodes and share them with multiple OPC UA clients running in the same Raspberry Pi led to session management overhead and considerably increased the average platform CPU utilization. This high session management overhead while managing the increased number of clients through individual TCP/IP channels drove the CPU to reachin saturation state, gradually raising the CPU temperature for a long time of operation and, in rare cases, causing unexpected sudden thermal shutdowns. However, this behavior was noticed when deploying the clients in individual stations instead of in the same Raspberry Pi. Such behavior presents a barrier to deploying the entire infrastructure with full functionalities serving a large number of OPC UA clients in the same resource-constrained Raspberry Pi, compared to the Intel NUC PC. In the future, this Python FreeOpcUa stack-based implementation will be refurbished with the Open62541 OPC UA protocol stack. This open-source full-stack OPC UA server–client implementation also involves a UA-TCP and UA-Binary transport scheme that has relatively low CPU demand, making it highly suitable for embedded systems [62]. Publish–subscribe-based application layer protocols such as AMQP, XMPP, and MQTT will be studied to integrate the middleware data with other popular cloud platforms such as Azure from Microsoft, BlueMix from IBM, and MindSphere from Siemens. Additional graphical plugins and data analysis tools will be included with the web service client to represent data in a more meaningful way. Furthermore, a multifactor authentication-based self-disposing dynamic token scheme will be implemented in the middleware request to perform more secure communication over the internet.

## Figures and Tables

**Table 1:** Key specifications of some popular OPC UA protocol stacks.

Criteria	Eclipse Milo	FreeOpcUa C++	FreeOpcUa Python	Node-Opc-Ua	OPC UA.NET	Open62541
Language	Java	C++	Python	JavaScript	C#	C
Licensing	Eclipse	LGPL	LGPL	MIT	RCL, MIT	MPLv2.0
Certificate	None	None	None	None	Yes	None
Implementation	Server and Client	Server and Client	Server and Client	Server and Client	Stack	Server and Client
UA-TCP UA-Binary	√	√	√	√	√	√
HTTPS UA-Binary	√				√	
HTTPS UA XML	√				√	
GetEndPoint	√	√	√	√	√	√
FindServer	√	√	√	√	√	√
AddNodes	√	√	√		√	√
AddReference	√	√	√		√	√
DeleteNodes	√		√		√	√
DeleteReference	√		√		√	√
Browse	√	√	√	√	√	√
Read	√	√	√	√	√	√
HistoryRead	√		√		√	
Write	√	√	√		√	√
Methods	√		√		√	√
CreateMonitoredItem	√	√	√	√	√	√
CreateSubscription	√	√	√	√	√	√
Publish	√	√	√	√	√	√
Basic128Rsa15	√		√	√	√	
Basic256	√		√	√	√	
Basic256Sha256	√		√	√	√	
Anonymous	√	√	√	√	√	√
Username Password	√		√	√	√	√
X.509 Certificate	√		√		√	
Platform	Linux, Windows	Linux, Windows	Linux, Windows	Linux, Mac, Windows	Windows	Linux, Windows

**Table 2:** List of key methods used to develop the middleware module.

Method No.	Method Name	Method Scopes
1	client.connect(Server Endpoint URI)	Connect with the server through the endpoint URI.
2	get_root_node().get_children()	Server address space objects under the root node.
3	get_object_node().get_children()	List of available variable nodes of a specified object.
4	get_node(node_id)	Get a node credentials via passed node id.
5	get_node(node_id).get_browse_name()	Get the variable node name via passed node id.
6	get_node(nodeid).get_value()	Read variable node contents via passed node id.
7	get_node(nodeid).set_value(Value)	Write value in a node via passed node id.

**Table 3:** Prescribed format to construct the web and cloud request.

Web Service:	http://192.168.0.194:8080/OPC?data=$Object1Name*Node1=Value1|...|Node N=Value N$
ThingSpeak:	https://api.thingspeak.com/y={}&fieldn=value n update?api_key={}&field1=value 1&…&field8=value 8
ThingSentral:	http://thingssentral.io:443/postlong?data=userid@Node1|Value1@......@Node N| Value N@

**Table 4:** List of command arguments scripted for the Telegram remote commander.

Commands	Command Scope in OPC UA Server
Commands?	Get the list of all the scripted commands.
Server Terminals?	Get all the server address space object node mames.
Server Tags?	Get all the OPC UA server variable node names.
Server nid?	Get all the OPC UA server variable node id.
ServerWritable	Get the list of OPC UA server writable variable nodes.
@Write.NodeName@Value	Write a value mentioned in the passed node name.
ClientDatalog	Get the Client Data Log file.
CloudDatalog	Get the Cloud Data Log file.
